# Deep Learning-Based and Python-Driven Construction and Application of a Mass Spectrometry Data Analysis Workflow: Taking Glucosinolates as an Example

**DOI:** 10.3390/metabo16040274

**Published:** 2026-04-17

**Authors:** Shangshen Yang, Siyu Jia, Peiyu Jia, Wenyu Xie, Xiaoming Wang

**Affiliations:** 1 Innovation Institute of Chinese Medicine and Pharmacy, Shandong University of Traditional Chinese Medicine, Jinan 250355, China; 2023111525@sdutcm.edu.cn (S.Y.); 2024111655@sdutcm.edu.cn (S.J.); 2023111527@sdutcm.edu.cn (W.X.); 2 Grade 2023 of Academy of Basic Medicine, Jining Medical University, Jining 272000, China; jiapeiyu@stu.mail.jnmc.edu.cn; 3 Experimental Center, Shandong University of Traditional Chinese Medicine, Jinan 250355, China; 4 Shandong Key Laboratory of Digital Traditional Chinese Medicine, Jinan 250355, China; 5 Shandong Province Cardiovascular Disease TCM Precision Treatment Engineering Research Centre, Jinan 250355, China; 6 Key Laboratory of Traditional Chinese Medicine Classical Theory, Ministry of Education, Jinan 250355, China

**Keywords:** UPLC-QE-MS/MS, mass data processing, automated compound screening, chemical structural characterization

## Abstract

Background: Radish seeds are our model on glucosinolates (GSLs), which is a class of secondary metabolites in medicinal plants of the Brassicaceae family. Multilayer perceptron (MLP) network is highly effective in the study of complex plants. This study came up with a smart plan through the Python language. Methods: First, we used the MLP network to pick out GSL precursor ions, running them through a deep learning filter. Next, we set up an automated screening system and looked at how standard chemicals break down. To speed things up, we created a scoring system that flagged promising compounds. After that, we built a tracer molecular network, basically connecting compounds according to how the plant makes them, which helped us label everything accurately. Finally, we brought in a math-based tool that pieces together different chemical parts to predict new GSL structures. Results: With this workflow, we annotated 195 glucosinolate-related compounds in radish seeds. That includes 86 regular GSLs, 34 malonyl products, 40 sinapoyl compounds, and 35 diglycosides. Among them, eight compounds were confirmed by comparison with authentic standards (retention time and MS/MS data), whereas the remaining compounds were tentatively annotated based on accurate mass measurements, diagnostic fragment ions, Tracer Molecular *Nnetworking*, and literature/database matching. In addition, 36 compounds were considered putatively novel derivatives pending further structural confirmation. Conclusions: This new approach reduces the time spent on determining chemicals in complicated samples. This can be done with other vegetables and medicinal herbs by researchers. It assists us in knowing the chemistry of plants in a deeper manner.

## 1. Introduction

The radish seed (*Raphanus sativus* L.) is a large crop of Brassicaceae and has been found to be rich in bioactive compounds [1]. These seeds are not only a source of basic nutrition, but also are highly beneficial to health [2]. Glucosinolates (GSLs) are one of the major advantages of it [3]. Radish seeds have specific flavor and biological power, which is attributed to GSLs. Structurally each GSL is made of a basic group consisting of 2-hydroxyimidosulfate cis-N-hydroxy and a 2-D-thioglucopyranoside molecule, but the side-chain substituents differ [4,5,6]. We classify them broadly into three categories: aliphatic, aromatic and indolic. It has been found out that GSLs and their products breakdown products are of significant importance in relation to cancer [7,8,9,10,11]. Due to this fact, GSL-enriched plants are promising candidates to new medicines and supplements. They also assist the plants to protect themselves and determine the taste of most of the vegetables [12]. Owing to their significance, we are in dire need of alternative means of locating and identifying GSLs in plants.

At present, the conventional isolation procedures of individual compounds are too slow and labor-intensive. Researchers typically resort to nuclear magnetic resonance (NMR) or mass spectrometry (MS) in order to detect structures [13,14]. NMR spectroscopy is a widely used analytical technique because it provides detailed structural information about chemical compounds and allows non-destructive analysis of samples, However, its sensitivity is generally lower than that of mass spectrometry-based techniques, which limits its ability to detect a large number of low-abundance components simultaneously [15]. Meanwhile, Liquid Chromatography-Tandem Mass Spectrometry (LC-MS/MS) would be far more effective in this endeavor. It has the ability to isolate compounds online and identify thousands of targets in a solitary procedure with 10 to 100 times the sensibility of NMR. In the case of GSLs, negative mode LC-MS/MS ([M-H]^−^) is a rich source of fragment information that is critical in the identification of known and unknown structures [16,17,18,19].

But the analysis of all this LC-MS/MS data is a huge burden. The classical workflow begins with the measurement of mass and prediction of formulas, yet complicated samples of plants typically generate excessively numerous candidate matches. Although such tools as MetFrag or CFM-ID are useful, they do not always address GSLs and may be imprecise [20,21,22]. Other researchers are employing a Diagnostic Ion Filtering (DIF) strategy in order to identify certain structural components, yet this also demands extensive hand work, which is time consuming and open to human error [23,24,25]. Even the modern, tools such as GNPS molecular networking are not able to work with samples of complex nature [26]; it tends to generate messy networks with an excess amount of redundant nodes and it is difficult to isolate particular GSL subtypes.

Data analysis has been made smarter and more flexible with the use of deep learning and Python programming (Python 3.11.9) [27,28,29,30]. Python is also easy to use and has a scalability that is ideal in being adapted to various kinds of chemicals. To address these bottlenecks in this work, we created an intelligent data-mining tool in Python. In this system, Mass Defect Filter (MDF) aided by a deep learning is more user-friendly in comparison with classical models. Another technology we developed is an Automated Diagnostic Ion Screening System where users are able to specify key fragments to lock in target compounds via a scoring system. In the end, we developed “GSL Tracer Molecular Networking” (TMN) to be able to visualize and mine information in a more efficient manner. This plan reduces the technical level of researchers and simplifies the mass spectrometry data significantly. We were able to apply this technique to map the GSLs of radish seeds and think that it can be expanded to map how we analyze other and more complicated plant systems in the future.

## 2. Materials and Methods

Radish seeds were purchased from Shandong Provincial Hospital of Traditional Chinese Medicine (Jinan, Shandong, China; batch number: 230502). 8 GSLs standards, including gluconapin, progoitrin, glucotropaeolin, glucoerucin, gluconasturtiin, glucoiberin, glucoraphenin, and glucobrassicin, were obtained from PhytoLab GmbH & Co. (Vestenbergsgreuth, Germany). Methanol, acetonitrile, and formic acid were purchased from Fisher Scientific (Thermo Fisher Scientific, Waltham, MA, USA). Stock standard solutions (0.50 mg/mL) were prepared by dissolving the standards in ultrapure water. Distilled water was purchased from Watsons Food and Beverage Co., Ltd. (Guangzhou, China).

Radish seeds were washed with distilled water, blotted dry, freeze-dried, ground into a fine powder using a grinder, and stored at −20 °C until analysis. For GSLs extraction, 10 g of sample powder was accurately weighed and mixed with 100 mL of 70% (*v*/*v*) methanol aqueous solution. We used an ultrasonic generator to extract the compounds for 30 min, repeating this step three times. After combining all the extracts, we concentrated them to dryness in a water bath at 60 °C. The resulting residue was dissolved in distilled water to prepare the final sample solution with a concentration of approximately 20 mg/mL. The sample solution was centrifuged at 12,000 rpm and 4 °C for 15 min. The supernatant was collected and filtered through a 0.22 μm microporous membrane prior to LC-MS analysis.

LC-MS and MS/MS data were obtained on a UHPLC Ultimate 3000 coupled with Q-Exactive Orbitrap-MS instrument (Thermo Fisher Scientific, San Diego, CA, USA). Chromatographic analysis was performed on an ACQUITY UPLC^®^ HSS T3 column (2.1 × 100 mm, 1.8 µm). The mobile phase consisted of A (water containing 0.05% formic acid) and B (acetonitrile containing 0.05% formic acid). The gradient was as follows: 0–5 min, 0% B; 5–9 min, 0–1% B; 9–10 min, 1–5% B; 10–12 min, 5–5% B; 12–14 min, 5–13% B; 14–17 min, 13–15% B; 17–19 min, 15–15% B; 19–22 min, 15–22% B; 22–29 min, 22–26% B; 29–31 min, 26–30% B; 31–33 min, 30–70% B; 33–35 min, 70–100% B; 35–40 min, 100% B; 40–40.5 min, 100–0% B; 40.5–45 min, 0% B. The column temperature was 30 °C, the flow rate was 0.2 mL/min, the injection volume was 5 μL, and the autosampler was maintained at a constant temperature of 15 °C.

The mass spectrometer was equipped with a heated electrospray ionization (HESI) source, and the following parameters were used: Sheath gas pressure: 45 arb; Auxiliary gas pressure: 10 arb; Spray voltage: 3.00 kV; Capillary temperature: 350 °C; Heater temperature: 350 °C. The samples were analyzed in Full-ms/ddMS^2^ mode, while the standards were analyzed in Target-SIM/ddMS^2^ mode. The mass range was set to *m*/*z* 80–1200 in negative ionization mode.

This study constructed a fully connected neural network model with a total of five layers, including four hidden layers. The input layer dimension was 4 (composed of the integer and decimal parts split from the characteristic M-H and m-H, where m–H represents the value obtained after removing the glucosyl–sulfated core from M–H), and the number of nodes in the hidden layers was 512, 256, 128, and 64 in sequence. A ReLU activation function was connected after each layer, and batch normalization and Dropout with a dropout rate of 0.01 were used for regularization. The output layer dimension was 2. In order to solve the issue of imbalance in the classes of data, the model used Focal Loss as a loss and was optimized using Adam optimizer. To balance the different classes, we resampled our training data using the SMOTETomek algorithm. In the training stage, we fixed the batch size of 64 and trained the model by 100 epochs. We used the loss and accuracy curve both in training and test sets to trace the progress in real time. We further made some attempts to normalize the parameters of the network by such means as batch normalization, Dropout, and L2 regularization. With these multi-dimensional constraints, which included saving optimal weights, we were able to use this to efficiently prevent overfitting (to ensure balanced and sound model performance).

To automate the screening of diagnostic ions in MS/MS, we designed a mass spectrometry data analysis program in Python 3.11.9, which is designed to assist in the analysis of data. The Python-based program employed a three-step parameter mechanism comprising a logic judgment system with AND/OR mode, a relative abundance parameter ranging from 0 to 1, and a default mass tolerance of ±5 ppm for precursor ion merging. These parameters can be adjusted by users according to the characteristics of the target compounds and the performance of different mass spectrometry instruments. The basic processing procedure had four steps. First, the program did the preprocessing of data and feature extraction of mass spectrometry data, the data was imported and parsing to extract the necessary data (mass-to-charge ratio (*m*/*z*), retention time (RT), charge state, as well as fragment ion properties). Second, diagnostic ions were filtered in the system based on logic criteria. The “AND” mode needed that all the required ions must have been matched, whereas the “OR” mode needed that one of the characteristic peaks required a match. To be confirmed as a valid diagnostic ion, candidate ions had to satisfy both the logical rules and the relative abundance settings simultaneously. After this, the program merged the screened precursor ions based on the assigned mass tolerance. Finally, the system exported the results into an Excel table that detailed the mass spectrometry ID, exact precursor mass, charge state, retention time, and the specific diagnostic ions with their relative abundances.

Based on the MS fragmentation rules of representative GSLs, a characteristic molecular networking tool running on Python (version 3.11.9) was developed. First, we used the open-source software MZmine 4.0.8 to process the UHPLC-MS/MS data of the extracts, and then the obtained feature quantification table (CSV format) and MS files (MGF format) were processed in Python (version 3.11.9). Further Python (version 3.11.9) processing includes the following steps: first, the MGF files (MS^2^ mass spectrometry information), which were processed by MZmine 4.0.8, were imported with CSV files (feature quantification tables). Second, the corresponding diagnostic ions and mass tolerance were input. If multiple or alternative characteristic ions exist, the logical relationship was input as “and” or “or”. The minimum abundance threshold was set. Finally, MGF files and optimized CSV files that match the GSL diagnostic ions and neutral losses were exported.

Glucosinolates consist of a basic glucosyl–sulfated core and a side chain, where the side chain is typically composed of multiple CH_2_ units. Based on this characteristic, we first classified the diagnostic ions and precursor ions obtained via deep learning (MDF). Subsequently, the precursor ions were subtracted by the GSL glucosyl–sulfated core (C_7_H_11_O_9_NS_2_, mass = 316.9881). The integer part of the resulting mass value was used as the *x*-axis, and the decimal part as the *y*-axis. Next, using the linear equation of CH_2_, the distance from each point to the line was calculated, ensuring that the lines connecting these points to the CH_2_ linear equation were parallel. To categorize these points, we focused on the integer parts of the mass values. We grouped any points that differed by multiples of 14 Da, as this specific gap indicated that the compounds were related by a difference in *n* CH_2_ units.

Construction of Deep Learning-Assisted MDF: The initial approach of the study was to determine the precursors of the target GSLs. An extensive database of mass spectrometry of *Raphanus sativus* L. seed was created by accessing the open databases, such as PubMed, PubChem, and SciFinder. The essential information that was incorporated in this process included chemical names, molecular formulas, and classifications. Also, the database contained diagnostic ions of characteristic identification and characteristic neutral loss fragments. Theoretical mass values of mass spectrometry ions for each component in the database were accurately calculated based on their chemical formulas. The basic dataset and extended dataset required for the artificial neural network were constructed: the training set included basic data (theoretical mass spectrometry ion mass values) and extended data (obtained by adding ±10 ppm deviations to the theoretical mass values). The test set followed a similar integration logic, but its extended data used values calculated with ±5 ppm deviations. Each dataset consisted of three parts: integrated theoretical mass spectrometry ion mass values of compounds, precise compound mass minus glucosinolate glucosyl–sulfated core mass, and labels. Subsequently, the model was trained using the datasets, and the trained model was loaded to perform classification and prediction of glucosinolates in the experimental dataset obtained via UHPLC-Q-Exactive Orbitrap-MS.

Automated Diagnostic Ion Screening System: This step aims to establish a mass spectrometry data mining method for glucosinolates based on their MS/MS fragmentation rules and diagnostic ions. A fragmentation characteristic library of glucosinolates was constructed by analyzing literature on glucosinolate fragmentation rules and combining the fragmentation patterns of glucosinolate standards. Diagnostic ions of glucosinolates were summarized by reviewing the relevant literature, and their relative abundance ranges were determined by referencing the fragmentation rules of standard substances. Based on the above fragmentation characteristics, multi-level screening conditions were set: AND-type diagnostic ions: *m*/*z* 96.9601 (relative abundance range: 0.8–1), *m*/*z* 74.9872, *m*/*z* 259.0129; OR-type diagnostic ions: *m*/*z* 79.9574, *m*/*z* 95.9517 (SO_4_^−^), *m*/*z* 274.9901 (C_6_H_11_O_8_S_2_^−^), *m*/*z* 241.0024; Precursor ion merging tolerance: ±5 ppm.

Design of GSLs Tracer Molecular Networking: Raw data were normalized using feature detection and alignment tools in MZmine 4.0.8 to be compatible with annotation tools. Briefly, for mass spectrometry detection: MS^1^ noise level: 1.0 × 10^4^; MS^2^ noise level: 0. Chromatograms were constructed using the ADAP chromatogram builder module with the following parameters: minimum time span was 0.05 min, minimum height was 1.0 × 10^5^, tolerance was 0.01. The constructed chromatograms were deconvoluted using the local minimum resolver with the following settings: chromatographic threshold = 96%, RT range = 0.05 min, minimum absolute height = 1.0 × 10^4^, minimum peak top/edge ratio = 1.7, peak duration range = 0.02~1.0 min. Isotope removal was performed using the isotope peak grouper algorithm (tolerance = 0.01, RT tolerance = 0.05 min). The join aligner and peak finder modules were used for feature alignment and gap filling. Based on fragmentation rules, the summarized diagnostic ions were input: Primary characteristic ions: *m*/*z* 96.95 and 74.98 (tolerance = 0.01 Da), minimum relative abundance = 0.5 (to increase target quantity while maintaining strictness for interference elimination); Secondary ions: *m*/*z* 259.0128, *m*/*z* 240.9657, *m*/*z* 192.0194 (mild threshold = 0.01 Da for accurate target identification). Finally, the optimized file was submitted to GNPS for mapping to characteristic molecular networks.

Substituent Combination: Four basic GSL glycosyl–sulfated core were set: [C_7_H_11_O_9_NS_2_]^−^ (*m*/*z* 316.9880 Da), [C_13_H_21_O_14_NS_2_]^−^ (*m*/*z* 479.0409), [C_10_H_13_O_12_NS_2_]^−^ (*m*/*z* 402.9885), and [C_18_H_21_O_13_NS_2_]^−^ (*m*/*z* 523.0460). After classification, the precise mass of each precursor ion was subtracted by the mass of the glycone moiety. Compositions of common substituents were calculated via permutation and combination, and the molecular formula of each compound was derived by adding the calculated substituent combination to the glycone moiety formula. Python (version 3.11.9) was used to call the PubChem API to search for relevant information (e.g., compound name, molecular weight, SMILES string) based on the molecular formula.

## 3. Results and Discussion

### 3.1. Deep Learning-Assisted MDF

**Figure 1:**
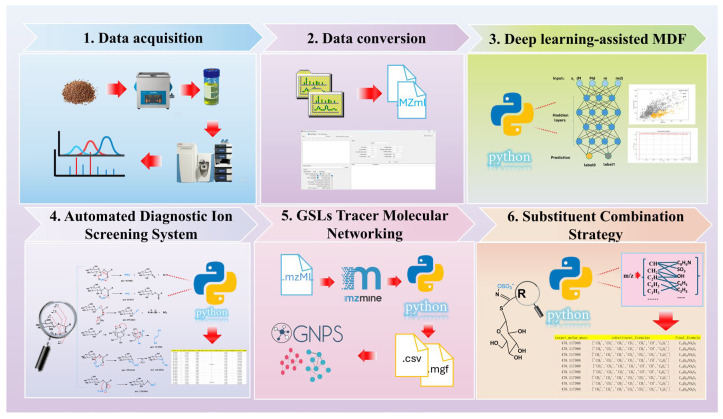
A comprehensive LC-MS/MS data analysis workflow for GSLs annotation.

**Figure 2:**
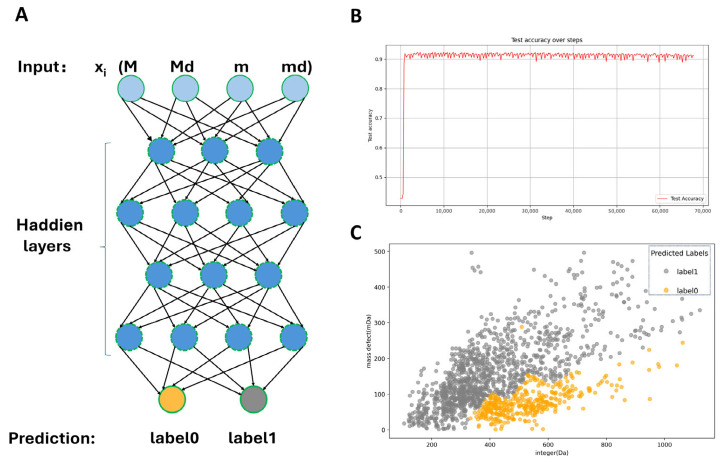
Construction of deep learning-assisted MDF. (**A**): Schematic diagram of the four-layer fully connected network algorithm (M = integer part of the accurate molecular weight (MW); Md = decimal part of the accurate MW; m = integer part of the accurate MW after subtracting the GSLs glucosyl–sulfated core; md = decimal part of the accurate MW after subtracting the GSLs glucosyl–sulfated core); (**B**): accuracy and training steps of the classification model; (**C**): deep learning classification results of precursor ions (0 represents GSLs; 1 represents other types of compounds).

We have developed a complete LC-MS/MS workflow of GSL annotation (Figure 1). To further improve the efficiency and accuracy of data interpretation in this workflow, machine learning-based models were introduced for automated feature extraction and annotation. Currently, commonly used artificial neural network architectures mainly include convolutional neural network (CNN) and fully connected neural network (FCNN). Compared with traditional machine learning methods (such as Support Vector Machines and Decision Trees), these feedforward neural networks exhibit distinct advantages in automatic feature extraction. A multilayer perceptron (MLP) is a type of fully connected neural network composed of multiple neuron layers, where each neuron is connected to every neuron in the previous layer (i.e., forming a fully connected structure). Typically consisting of an input layer, hidden layers, and an output layer, MLPs can learn complex mapping relationships through nonlinear activation functions, making them widely applicable to tasks such as classification and regression. For the low-dimensional feature dataset (number of samples N = 1493, feature dimension D = 32), an MLP suitable for low-dimensional classification tasks was employed for training. In this study, a build model of precursor ion categories was formed on the basis of FCNN model. The number of layers of the network architecture is five fully connected layers (Figure 2A) (i.e., input layer, four hidden layers, and an output layer). The generalization capability of the model was maximized by increasing the size of the training set and the model performance was eventually tested on an independent test set. The classification performance of the MLP model on the independent test set was evaluated using precision, recall, F1-score. As shown in Figure S3 in the Supplementary Materials, the model exhibited balanced and reliable classification performance, supporting its effectiveness for precursor ion screening. In addition, Figure S4 in the Supplementary Materials presents the explainable AI results of the model. The SHAP summary plot and permutation feature importance analysis further revealed the relative contributions of the four input features to model prediction. Among them, M_decimal (the decimal part of the accurate molecular weight) showed the greatest contribution, as evidenced by both the widest SHAP value distribution and the largest accuracy drop in the permutation feature importance analysis. M_integer and m_integer also made substantial contributions and showed comparable importance, whereas m_decimal had the smallest effect on model output. These results indicate that both the integer and decimal information derived from precursor-ion-related mass features contribute to GSL-related precursor ion screening, with the decimal part of the original accurate molecular weight playing the most influential role.

The model achieved the test set classification accuracy of 91.03% after 70,000 training iterations (Figure 2B). The network employed a dual-label prediction system, which established the two most likely categories of each precursor ion and the particular probability. In a practical test, the model was able to handle 1493 precursor ions within a few minutes. It led to the identification of 676 ions related to glucosinolate compounds being successful (Figure 2C). Experimental results demonstrate that this classification model can significantly improve the screening efficiency of precursor ions, providing a reliable data foundation for subsequent analyses.

### 3.2. Automated Diagnostic Ion Screening System

**Figure 3:**
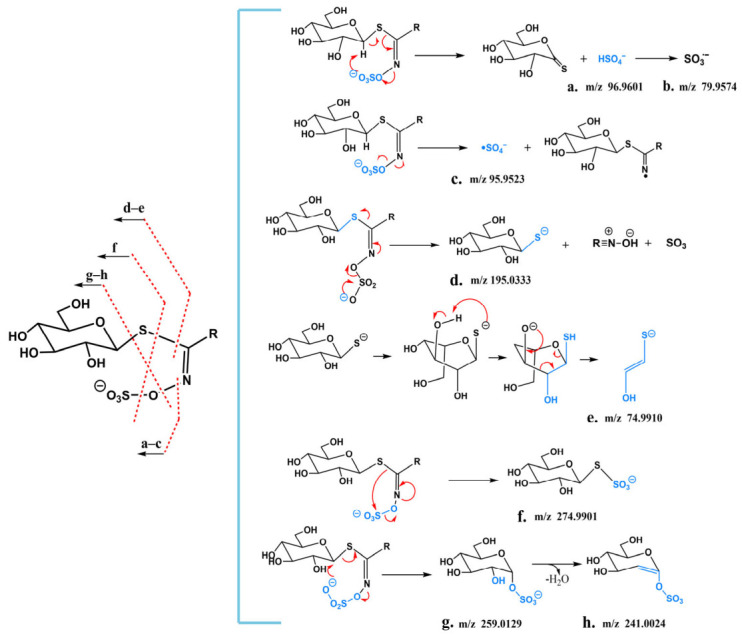
Characteristic diagnostic ions and their fragmentation patterns. (**a**–**h**) Diagnostic ions: (**a**) HSO_4_^−^ (*m*/*z* 96.9601); (**b**) SO_3_^−^ (*m*/*z* 79.9574); (**c**) SO_4_^−^ (*m*/*z* 95.9517); (**d**) *m*/*z* 195.0333; (**e**) *m*/*z* 74.9910; (**f**) *m*/*z* 274.9901; (**g**) *m*/*z* 259.0129; (**h**) *m*/*z* 241.0024.

To establish a strategy for the rapid discovery and systematic identification of GSLs, it is essential to comprehensively understand their characteristic fragmentation pathways. Therefore, LC–MS analysis was first performed on a mixture of eight GSL standards, and the corresponding total ion chromatogram (TIC) is shown in Figure S1; meanwhile, LC–MS analysis of radish seed samples was conducted to obtain the overall ion chromatogram (Figure S6). Based on the fragmentation rules summarized from the relevant literature and the MS/MS behaviors of the authentic standards analyzed in this study, ions were first classified as glucosinolate-related ions. These ions were then assigned to specific structural subclasses according to the corresponding subclass-specific fragmentation rules, including characteristic fragment ions and neutral loss patterns (Figure 3). According to their fragmentation rules, the MS^n^ fragmentation patterns of GSLs and their characteristic diagnostic ions can be classified into two categories, namely glycone-derived and side chain substituent-derived. One of the major fragment ions produced by all GSLs is *m*/*z* 96.9601 (HSO_4_^−^). Experiments confirmed that the hydrogen atom in this sulfate group originates from the glycosyl moiety, with the hydrogen atom at the C-1 position being the most likely contributor. It is speculated that its formation mechanism is achieved through a concerted elimination reaction of two neutral molecules, forming an eight-membered ring transition state structure (Figure 3a).

Diagnostic ions generated by GSLs also include *m*/*z* 79.9574 (SO_3_^−^) and *m*/*z* 95.9517 (SO_4_^−^). As shown in Figure 3b, *m*/*z* 79.9574 (SO_3_^−^) can be formed by the loss of a hydroxyl radical (-OH·) from *m*/*z* 96.9601 (HSO_4_^−^). According to relevant studies on the fragmentation mechanism of organic N-sulfate anions, the formation of the *m*/*z* 95.9517 (SO_4_^−^) fragment ion peak results from the direct homolysis of the N-O bond in the glucosinolate anion, as illustrated in Figure 3c.

*m*/*z* 74.9872 (C_2_H_3_OS^−^) and *m*/*z* 195.0333 (C_6_H_11_O_5_S^−^) are important glycone substituent-derived diagnostic ions. First, GSLs lose RCNSO_3_ from the thioglucose and sulfonated oxime moieties to form the characteristic diagnostic ion *m*/*z* 195.0333 (C_6_H_11_O_5_S^−^), as shown in Figure 3d. A six-membered transition state is more easily formed in the boat conformation. The negative charge on the sulfur atom is redistributed to the 3-position hydroxyl group through proton transfer in the six-membered ring transition state, generating its characteristic sulfide product ion *m*/*z* 74.9872 (C_2_H_3_OS^−^) (Figure 3e).

In addition, complex intramolecular rearrangements commonly occur during the formation of glycone substituent-derived ions of GSLs, characterized by the migration of the electronegative sulfate group to the thioglucose, resulting in the production of numerous specific fragment ions. These include *m*/*z* 274.9901 (C_6_H_11_O_8_S_2_^−^) (Figure 3f), *m*/*z* 259.0129 (C_6_H_11_O_9_S^−^), and *m*/*z* 241.0024 (C_6_H_9_O_8_S^−^) (Figure 3g). Their fragmentation pathways are as follows: *m*/*z* 259.0129 (C_6_H_11_O_8_S_2_^−^) loses the hydroxyl group at the 2-position of the glucoside and a molecule of H_2_O to generate the diagnostic ion *m*/*z* 241.0024 (C_6_H_9_O_8_S^−^) (Figure 3h). Furthermore, there is a set of fragment ions (R) with side chain structural characteristics, corresponding to R-CHONS, R-C_2_HO_4_NS, R-CHO_5_NS, and R-CHO_4_NS_2_, respectively, which are formed by the heterolytic cleavage of the C-S bond in thioglucose.

For the GSLs diagnostic ions identified above, a scoring rule was designed. GSLs’ molecular structures contain sulfate groups, which readily form [M-H]^−^ peaks in negative ion electrospray ionization MS. Among them, the product ion *m*/*z* 96.9601 exhibits a strong response in MS and is regarded as a key characteristic ion. It was set as an AND logic condition with a relative abundance range of 0.8–1. However, considering that most organic sulfate anions also form this anion through a six-membered cyclic syn-elimination mechanism, to improve the accuracy of scoring, the important characteristic ion *m*/*z* 74.9872 (produced during the fragmentation of glycone-derived fragments) and the rearrangement-derived characteristic ion *m*/*z* 259.0129 were also set as AND logic conditions. A base score of 10 is awarded if the AND logic conditions are met, and an additional score of 10 is given if the relative abundance range is satisfied. Additionally, *m*/*z* 79.9574, *m*/*z* 95.9517 (SO_4_^−^), *m*/*z* 274.9901 (C_6_H_11_O_8_S_2_^−^), and *m*/*z* 241.0024 were set as OR logic conditions, with 10 points awarded for each identified diagnostic ion.

### 3.3. GSLs Tracer Molecular Networking

**Figure 4:**
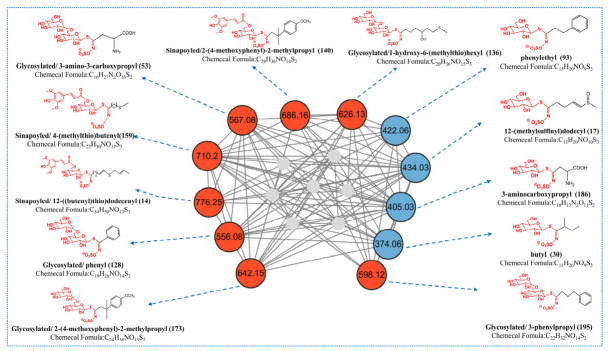
GSLsTracerMN (blue nodes represent known GSLs, red nodes represent unknown sinapoylated or glycosylated GSLs, and gray nodes represent unmatched compounds).

Traditional molecular networks construct nodes by calculating the cosine similarity of user-uploaded MS/MS data and then comparing the mass spectral similarity among different samples. Each node represents a mass spectral feature, and the edges between nodes denote a network of high similarity between mass spectra. Due to the large number and complexity of plant compounds, the sole use of molecular networks results in numerous redundant nodes. Therefore, based on the MS fragmentation rules of GSLs in representative cruciferous vegetables, we proposed a Python-based method, GSLsTracerMN, for processing MS data via molecular networking. Briefly, after processing the data with MZmine 4.0.8, we set the corresponding characteristic diagnostic ions and their abundances according to the MS/MS fragmentation rules of GSLs, and submitted the files processed by Python (version 3.11.9) to GNPS (https://gnps.ucsd.edu/ProteoSAFe/static/gnps-splash.jsp, accessed on 14 April 2026) for mapping to GSLsTracerMN. As shown in one cluster of compounds (Figure 4), the GSLsTracerMN consists of 19 nodes, which focus on compounds of specific GSL structural types of interest. Compared with the general analysis method of FBMN, GTMN removes redundant nodes more effectively. Interestingly, after detailed analysis of the MS/MS spectra of relevant nodes, we identified not only commonly known GSL backbones (represented by blue nodes in Figure 4) but also unknown glycosylated GSLs and sinapoylated GSLs. For further comparison, the molecular network generated by conventional FBMN is shown in Figure S5, and the quantitative comparison of network metrics between conventional FBMN and GSLsTracerMN is summarized in Table S1 in the Supplementary Materials. Compared with conventional FBMN, GSLsTracerMN markedly reduced redundant/background nodes and improved clustering clarity for GSL-related compounds.

### 3.4. Structural Annotation of GSLs

**Figure 5:**
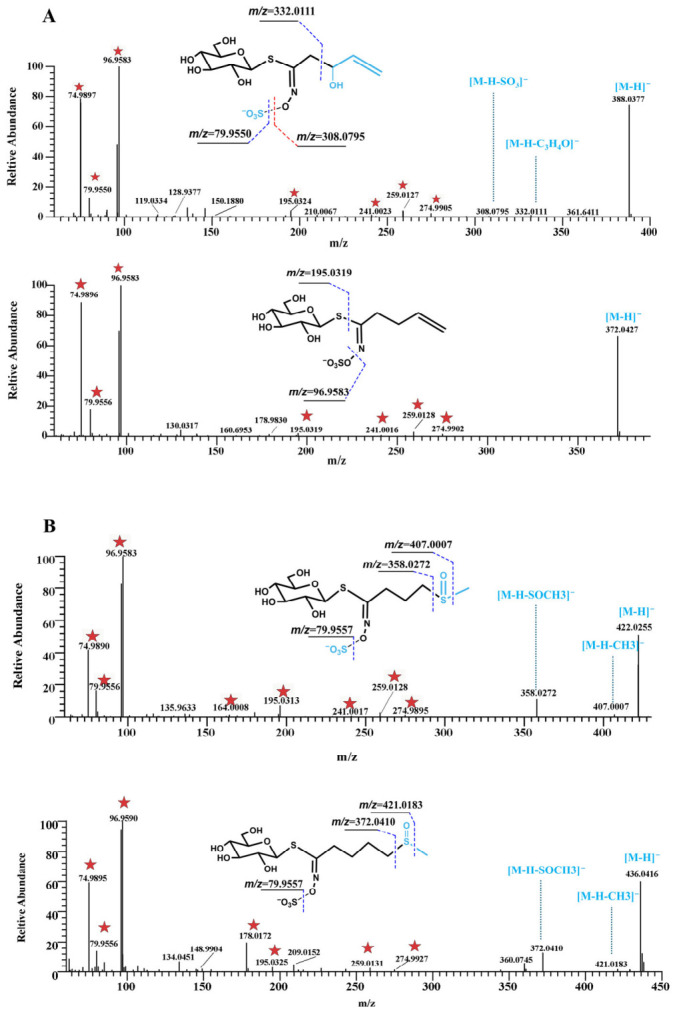
MS/MS spectra and fragmentation pathways of representative GSLs. (**A**) Compounds **12** and **20**; (**B**) Compounds **7** and **18**. Red stars indicate the characteristic diagnostic ions of GSLs.

In the negative ion mode, the optimized LC–MS spectra of the samples and reference substances are shown in Figure S1 in the Supplementary Materials. GSLs are generally classified into three major categories: aliphatic, aromatic, and indolic GSLs, with aliphatic GSLs further subdivided into alkyl, methylthio, sulfinyl, and sulfonyl glucosinolates. In this study, a total of 195 compounds were annotated according to the above workflow, and their retention times, molecular formulas, and mass spectrometric data are summarized in Table S2. The proposed structures of the characterized GSLs are shown in Figure S2. Among them, eight compounds were confirmed using authentic standards, whereas the assignments of the remaining compounds, especially the malonylated, glycosylated, and sinapoylated derivatives, were based mainly on accurate mass measurements, characteristic MS/MS fragmentation behavior, diagnostic ions, neutral loss patterns, and literature comparison, and should therefore be regarded as tentative annotations. All identified annotated compounds in the mass spectra exhibited characteristic fragmentation rules of GSLs, such as glycosidic bond cleavage and the generated core skeleton diagnostic ions, and the specific identification process is as follows:

Figure 5 presents representative MS/MS spectra and fragmentation pathways of different subclasses of GSLs, illustrating the key diagnostic ions and characteristic neutral losses used for structural annotation.

Alkyl GSL: Compounds **12** (*m*/*z* 388.0377) and **20** (*m*/*z* 372.0427) were taken as examples (Figure 5A). Both showed the characteristic diagnostic ions of GSLs, including *m*/*z* 74.9894, 96.9587, 259.0126, and 274.9890 [31]. Compound **12** generated a fragment ion at *m*/*z* 332.0111 by the loss of C_3_H_4_O (56.0266 Da). Compared with Compound **12**, Compound **20** was 16 Da lower in molecular weight and eluted later, which is consistent with the reported retention behavior of related GSLs [32].

Sulfinyl GSL: Compounds **7** (*m*/*z* 422.0255) and **18** (*m*/*z* 436.0416) differed by 14 Da, corresponding to one CH_2_ unit (Figure 5B). In addition to the common GSL diagnostic ions, sulfinyl glucosinolates typically showed characteristic neutral losses of CH_3_ (15.03235 Da) and CH_3_SOH (63.998 Da). For Compound **21** (*m*/*z* 450.0565, 9.91 min), a rearrangement ion at *m*/*z* 192.0336 was also observed, supporting its structural assignment [33].

Benzene-ring-containing GSLs: Compound **93** (*m*/*z* 422.0582) was taken as a representative example (Figure S7A). Its MS/MS spectrum contained the common diagnostic ions of GSLs, together with a fragment ion at *m*/*z* 342.0981 corresponding to [M−H−SO_3_]^−^. A fragment ion at *m*/*z* 180.0464 was also observed, which may result from intramolecular attack of the negative ion on the sulfonic acid group at C1 of the glucosyl moiety [34].

Indole GSLs: Compounds **72** (*m*/*z* 447.0535) and **132** (*m*/*z* 463.0494, 23.10 min) were selected as representative indole GSLs (Figure S7B). Both exhibited the characteristic diagnostic ions of GSLs. Compared with Compound **132**, Compound **72** showed a later retention time, in agreement with the chromatographic behavior of this subclass. In addition, Compound **132** produced several characteristic fragment ions at *m*/*z* 267.0083, 239.9963, 170.0476, and 160.0393, which provided important evidence for structural interpretation of the indole side chain [35].

Malonyl-substituted GSLs: Representative malonyl-substituted GSLs are shown in Figure S7C. For Compound **150** (*m*/*z* 488.0906), the fragment ions at *m*/*z* 444.0977 and 402.0866 were assigned to the neutral losses of CO_2_ (43.9929 Da) and C_3_H_2_O_3_ (86.0040 Da), respectively, while the ion at *m*/*z* 384.0763 was likely formed by further loss of H_2_O. For Compound **34** (*m*/*z* 520.0253, 14.23 min), the fragment ion at *m*/*z* 461.0145 indicated the loss of C_2_H_3_O_2_, and the ion at *m*/*z* 504.9996 suggested an additional methyl loss from the side chain.

Glycosylated and sinapoylated GSLs: As shown in Figure S7D, Compound **3** (*m*/*z* 584.0773) primarily lost C_6_H_11_O_5_ to form a fragment ion at *m*/*z* 421.0662, followed by a secondary fragment ion at *m*/*z* 403.0575, supporting its assignment as a glycosylated GSL. In addition, Compound **118** (*m*/*z* 642.0997,) exhibited characteristic sinapoyl-related ions at *m*/*z* 223.0603, 205.0500, and 190.0263, indicating the presence of a sinapoyl substituent on the glycosyl moiety.

Although the present study enables high-throughput annotation of GSLs, most compound assignments are based on MS/MS data and should be regarded as tentative, particularly because the differentiation of isomeric or closely related compounds remains challenging in LC–MS/MS-based annotation. In this study, such cases were evaluated using multiple complementary criteria, including authentic standard comparison, chromatographic retention behavior, and characteristic MS/MS fragmentation patterns. However, for compounds with highly similar structures and fragmentation behaviors, unambiguous differentiation was still difficult based solely on LC–MS/MS data, and these were therefore conservatively reported as tentative annotations or designated as “or isomer” in Table S2. Although NMR spectroscopy is highly powerful for distinguishing isomeric structures, it generally requires isolated single compounds. Comprehensive structural confirmation in complex matrices still relies on the integration of multiple orthogonal approaches, including improved chromatographic separation and ion mobility analysis for enhanced resolution, as well as NMR spectroscopy for definitive structural elucidation, particularly for novel, structurally modified, or isomeric GSLs.

## 4. Conclusions

GSLs, as the main active chemical components of cruciferous plants, have good pharmacological effects. At present, there is limited understanding of the structures of GSLs-type chemical components, and it is urgent to introduce AI data processing methods to discover their new skeletons and components. Based on UHPLC-Q-Exactive Orbitrap-MS, this study developed an intelligent workflow for the comprehensive annotation of GSLs compounds in radish seeds, a cruciferous vegetable. This workflow used Python (version 3.11.9) to successfully combine deep learning with MDF for precursor ion screening, utilized AND and OR logic combined with relative abundance thresholds to set diagnostic ions for scoring, set multiple parent nucleus substituents according to the structure of GSLs to calculate possible substituent compositions, and adopted multiple mass spectrometry identification strategies such as Tracer Molecular Networking technology with AND and OR logic combined with relative abundance thresholds to remove redundant nodes, thereby realizing the comprehensive analysis of GSLs chemical components. All identification work can complete the rapid analysis of thousands of chemical components in only a few hours, which improves the accuracy and speed of annotation results. This intelligent and customized workflow enriches our understanding of the chemical components of cruciferous crops and is worthy of promotion and application in the comprehensive characterization and chemical annotation of chemical components, such as other plants and natural products. Nevertheless, the effectiveness of this workflow is still influenced by the completeness of the fragmentation library and the coverage of external database resources, such as PubChem. As a result, rare, previously unreported, or insufficiently represented compounds may remain undetected or only be tentatively annotated.

## Data Availability

The original contributions presented in this study are included in the article (Supplementary Materials). Further inquiries can be directed to the corresponding author.

## Supplementary Materials

The following supporting information can be downloaded at: https://www.mdpi.com/article/10.3390/metabo16040274/s1, Figure S1: TIC of 8 standards under Target-SIM/ddMS2 mode. 1: glucoiberin (compound **7**), 2: progoitrin (compound **11**), 3: glucoraphanin (compound **18**), 4: gluconapin (compound **20**), 5: glucotropaeolin (compound **56**), 6: glucoerucin (compound **61**), 7: glucobrassicin (compound **72**), 8: gluconasturtiin (compound **93**); Figure S2: The proposed structures of 195 characterized GLSs; Figure S3: Classification report heatmap of the MLP classifier on the independent test set; Figure S4: Explainable AI analysis of the MLP model for precursor ion screening. (A) SHAP summary plot. (B) Permutation feature importance analysis based on accuracy drop. M_integer: integer part of the accurate molecular weight; M_decimal: decimal part of the accurate molecular weight; m_integer: integer part of the accurate molecular weight after subtracting the GSL glucosyl–sulfated core; m_decimal: decimal part of the accurate molecular weight after subtracting the GSL glucosyl–sulfated core; Figure S5: Molecular network generated by conventional FBMN. Red nodes represent glucosinolate-related features, while gray nodes represent other background or non-target features in the network; Figure S6: TIC of radish seed extracts from LC-MS analysis, with the major peaks annotated; Figure S7: MS/MS spectra and fragmentation pathways of representative GSLs. (A) Compound **93**; (B) Compounds **72** and **132**; (C) Compounds **34** and **150**; (D) Compounds **3** and **118**; Table S1: Comparison of network metrics between conventional FBMN and GSLsTracerMN; Table S2: The chromatographic and mass spectral data of 195 compounds were characterized by UHPLC-Q-Exactive Orbitrap-MS.

## Abbreviations

The following abbreviations are used in this manuscript:

MLP	Multilayer Perceptron
GSL	Glucosinolate
NMR	Nuclear Magnetic Resonance
MS	Mass Spectrometry
LC-MS	Liquid Chromatography-Tandem Mass Spectrometry
DIF	Diagnostic Ion Filtering
MDF	Mass Defect Filter
TMN	Tracer Molecular Networking
HESI	Heated Electrospray Ionization
RT	Retention Time
CNN	Convolutional Neural Network
FCNN	Fully Connected Neural Network
TIC	Total Ion Chromatogram
