# Supplementary material for: Deep Learning-Based and Python-Driven Construction and Application of a Mass Spectrometry Data Analysis Workflow: Taking Glucosinolates as an Example
Source: Metabolites. 2026 Apr 17;16(4):274. doi: 10.3390/metabo16040274 (PMC13118452; doi:10.3390/metabo16040274)
Supplement: Supplementary file 1 [file metabolites-16-00274-s001.zip › metabolites-4226628-supplementary.pdf]

Supplementary Materials S1 Supplementary Data.

Supplementary Materials S1.1

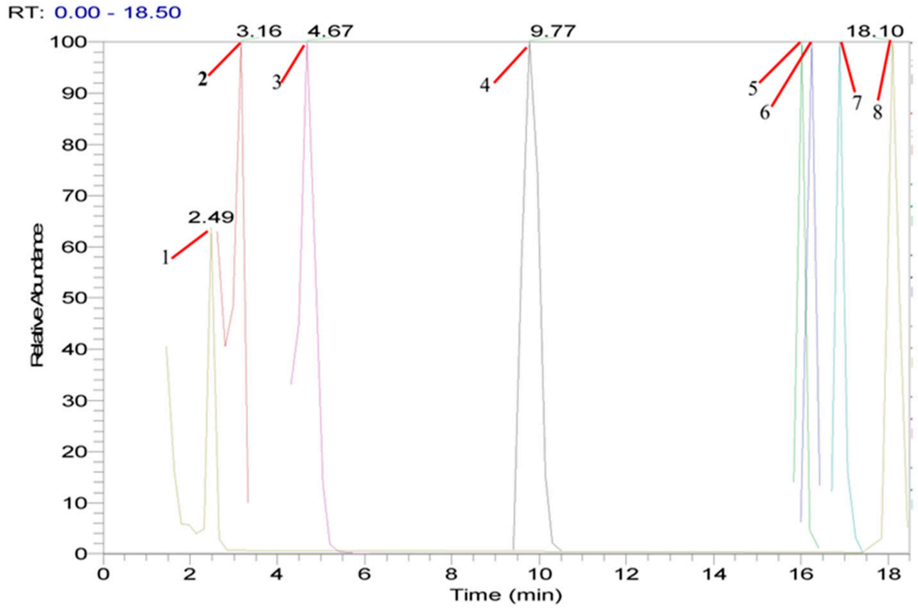

Figure S1. TIC of 8 standards under Target-SIM/ddMS2 mode;. 1: glucoiberin(compound 7), 2: progoitrin(compound 11), 3: glucoraphanin(compound 18), 4: gluconapin(compound 20), 5: glucotropaeolin(compound 56),6: glucoerucin(compound 61), 7: glucobrassicin(compound 72), 8:gluconasturtiin(compound 93).

Supplementary Materials S1.2

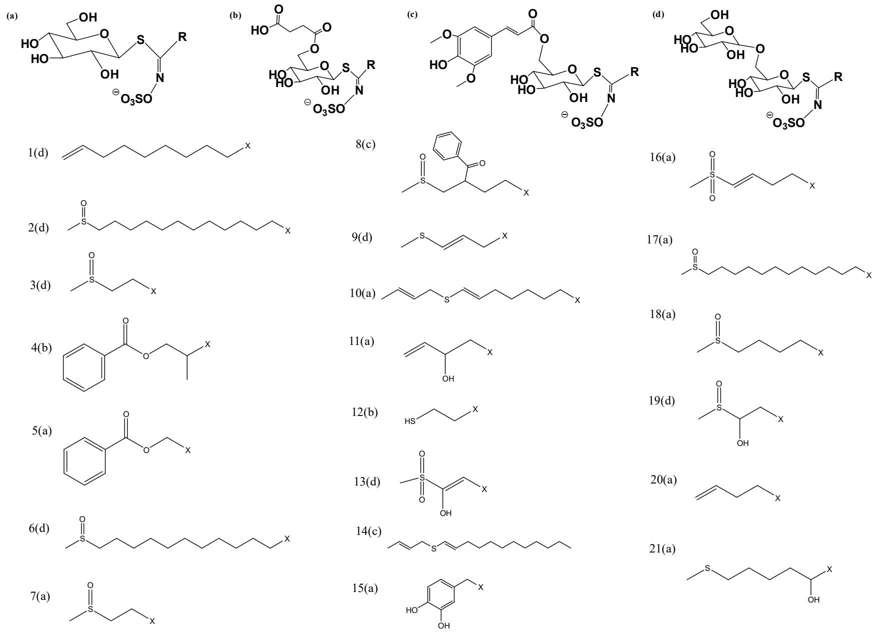

22(a), 23(a), 24(b), 25(b), 26(a), 27(a), 28(a), 29(c), 30(a)

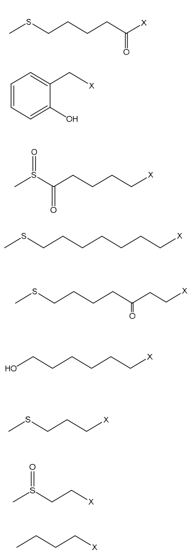

31(a), 32(a), 33(b), 34(b), 35(a), 36(c), 37(a), 38(a)

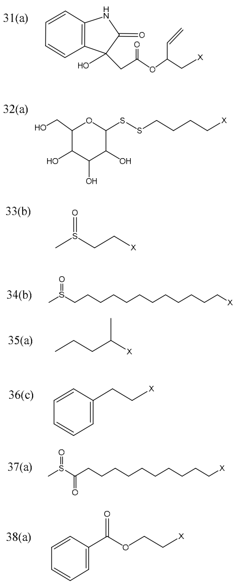

39(a), \40(a), 41(b), 42(b), 43(d), 44(a), 45(b), 46(a), 47(a), 48(b)

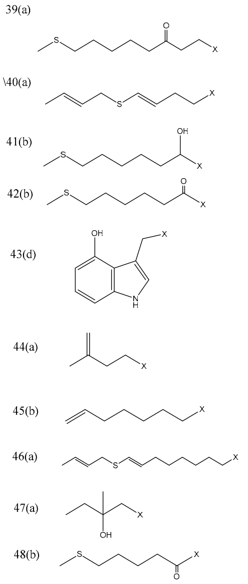

49(b), 50(a), 51(b), 52(a), 53(d), 54(d), 55(a), 56(a)

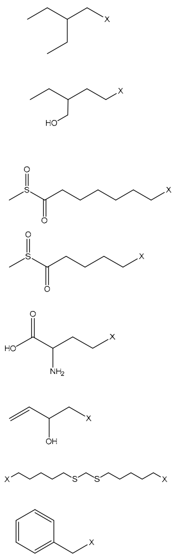

57(d), 58(a), 59(a), 60(a), 61(a), 62(c), 63(b), 64(b), 65(b), 66(c), 67(c)

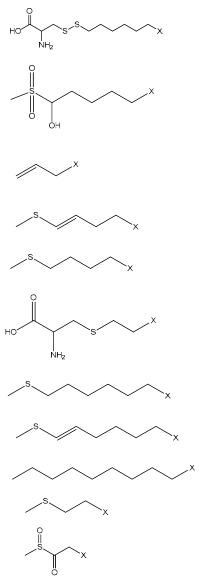

68(b), 69(a), 70(b), 71(d), 72(a), 73(c), 74(c), 75(a)

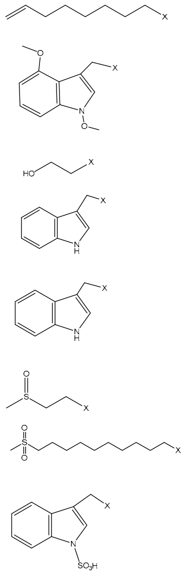

76(c), 77(c), 78(b), 79(d), 80(b), 81(c)

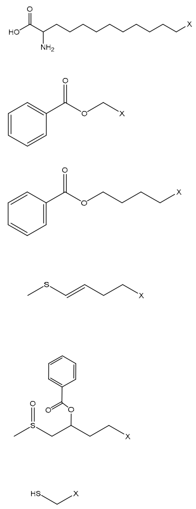

82(a), 83(a), 84(a), 85(d), 86(a), 87(a), 88(a), 89(a), 90(d)

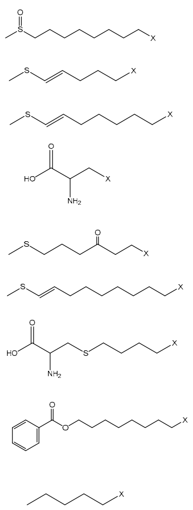

91(a), 92(a), 93(a), 94(c), 95(d), 96(a), 97(a), 98(a)

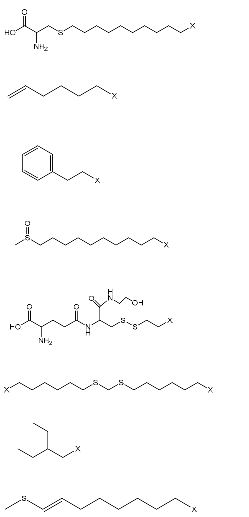

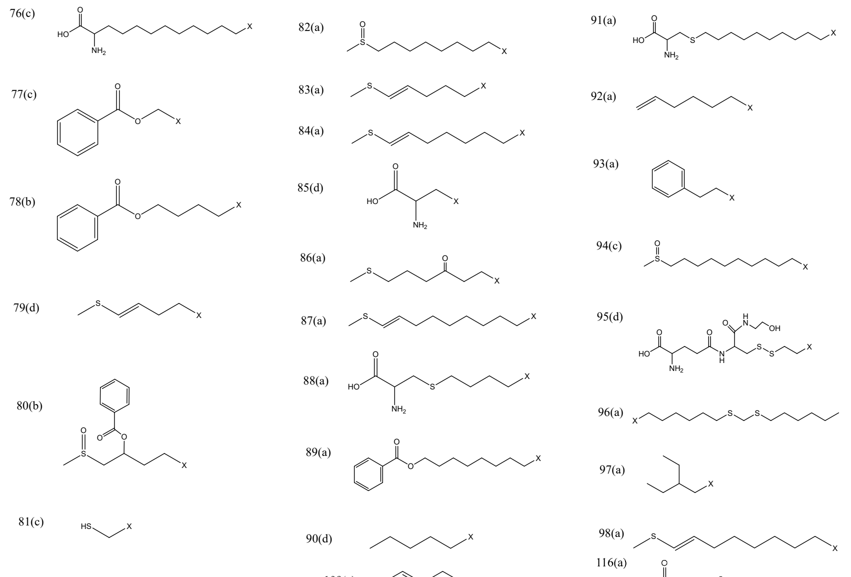
76(c)
82(a)
91(a)
77(c)
83(a)
92(a)
84(a)
93(a)
78(b)
85(d)
86(a)
94(c)
79(d)
87(a)
95(d)
88(a)
80(b)
96(a)
89(a)
97(a)
81(c)
90(d)
98(a)

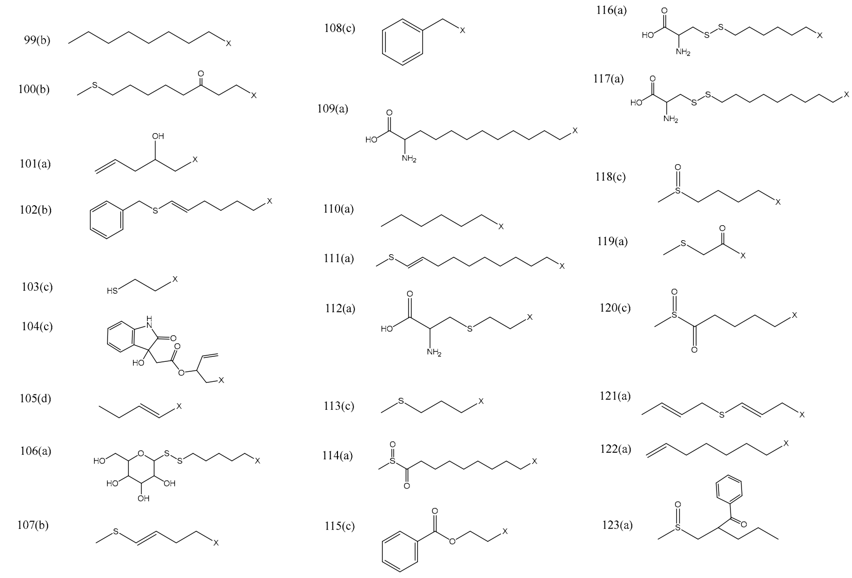
99(b)
108(c)
116(a)
100(b)
117(a)
109(a)
101(a)
118(c)
102(b)
110(a)
119(a)
111(a)
103(c)
104(c)
112(a)
120(c)
105(d)
113(c)
121(a)
106(a)
114(a)
122(a)
107(b)
115(c)
123(a)

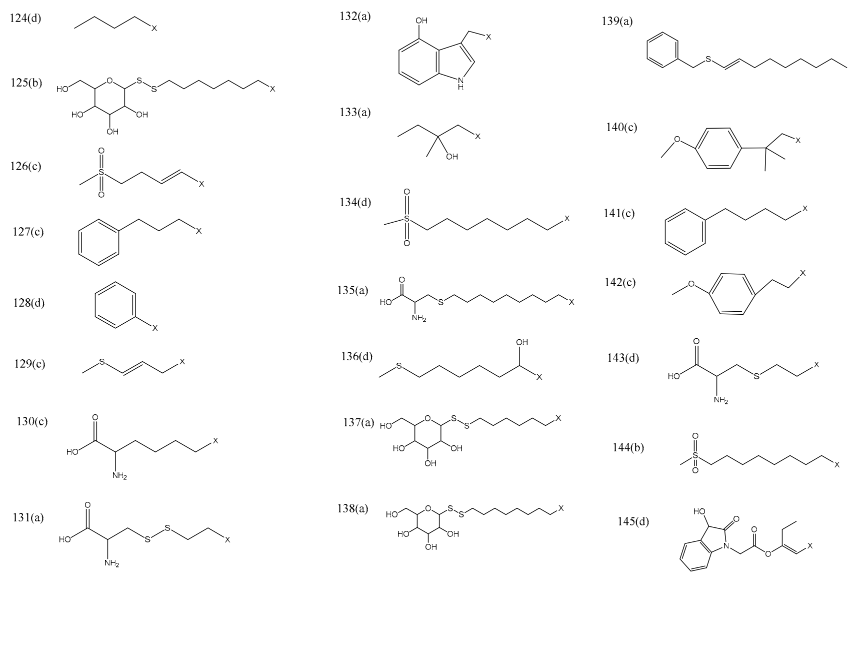
124(d)
132(a)
139(a)
125(b)
133(a)
140(c)
126(c)
134(d)
141(c)
127(c)
128(d)
135(a)
142(c)
129(c)
136(d)
143(d)
130(c)
137(a)
144(b)
131(a)
138(a)
145(d)

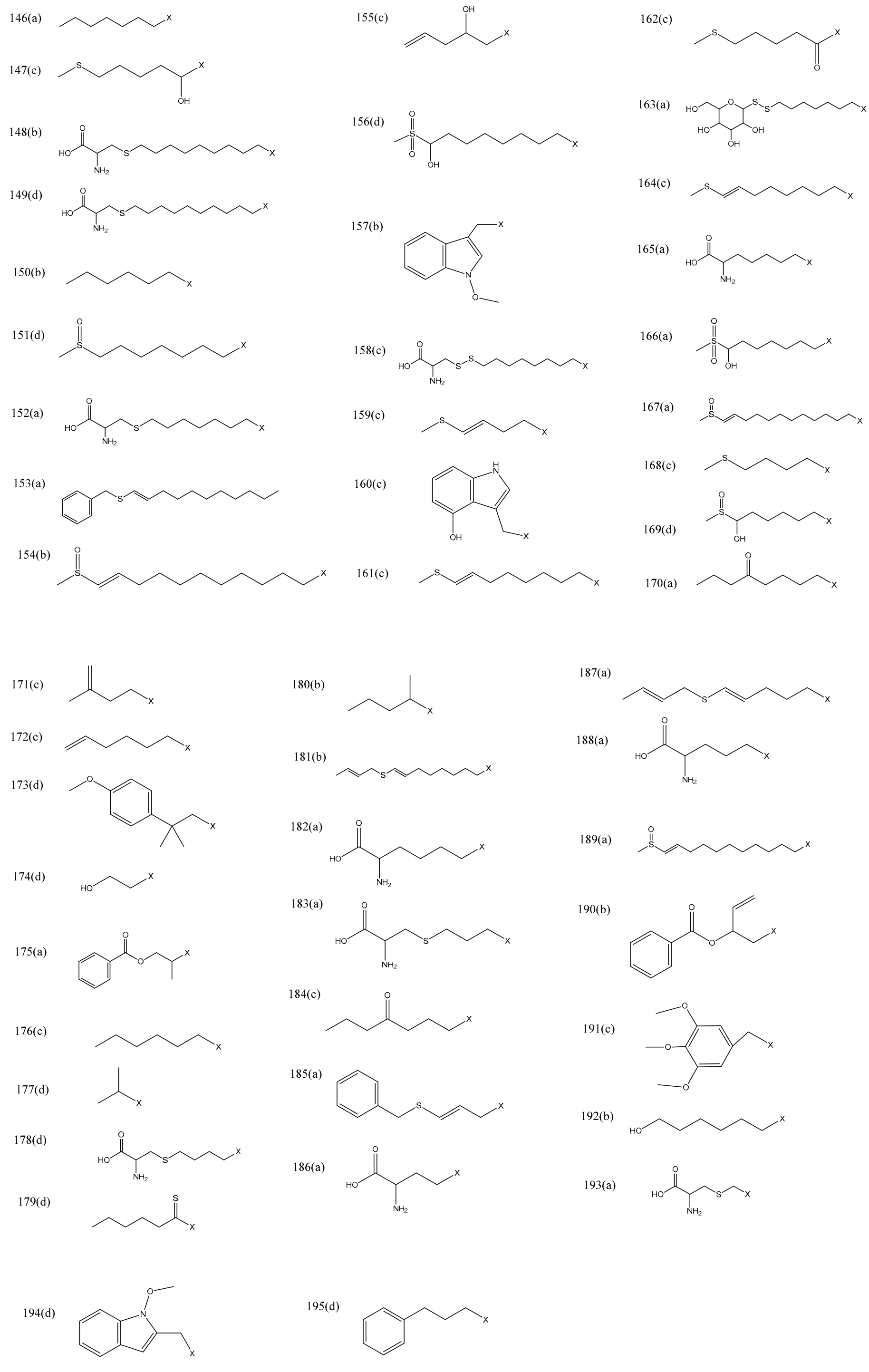

Figure S2. The proposed structures of 195 characterized GLSs.

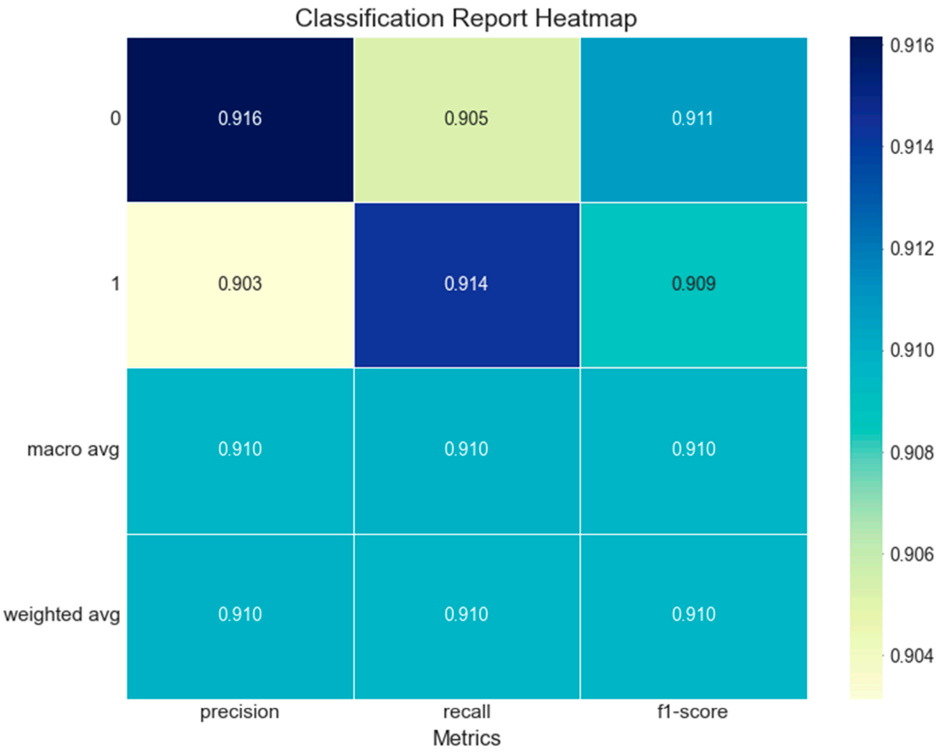

Figure S3. Classification report heatmap of the MLP classifier on the independent test set.

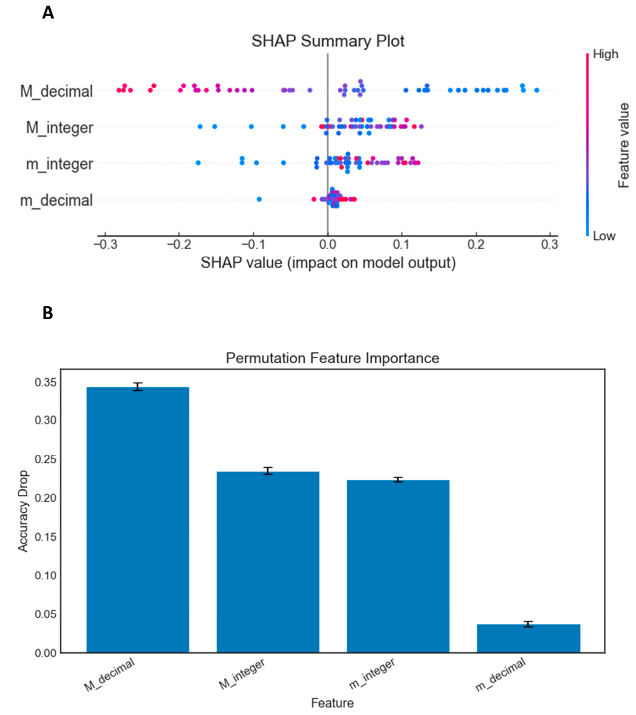

Figure S4 . Explainable AI analysis of the MLP model for precursor ion screening. (A) SHAP summary plot. (B) Permutation feature importance analysis based on accuracy drop. M_integer: integer part of the accurate molecular weight; M_decimal: decimal part of the accurate molecular weight; m_integer: integer part of the accurate molecular

weight after subtracting the GSL glucosyl–sulfated core; m_decimal: decimal part of the accurate molecular weight after subtracting the GSL glucosyl–sulfated core.

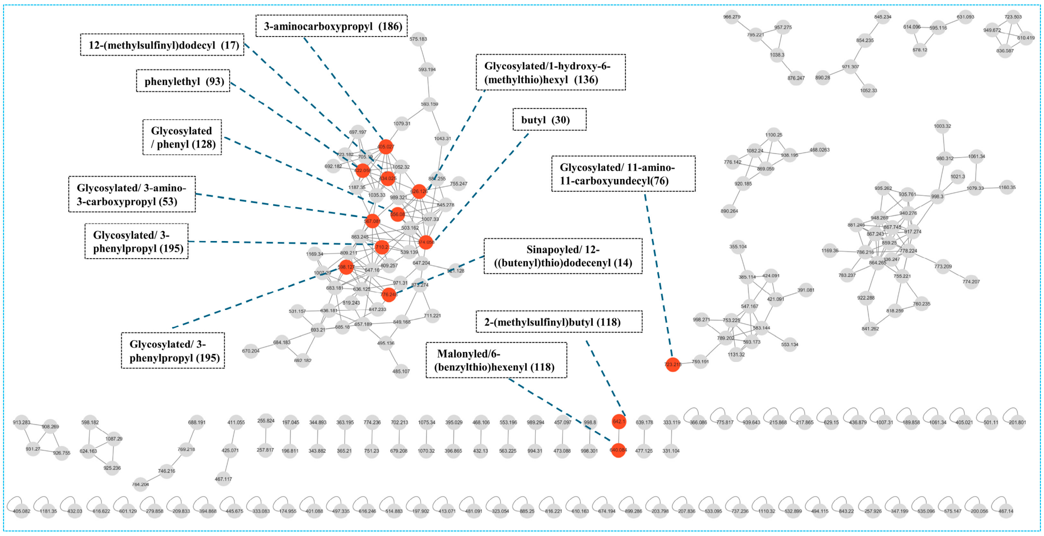

Figure S5. Molecular network generated by conventional FBMN. Red nodes represent glucosinolate-related features, while gray nodes represent other background or non-target features in the network.

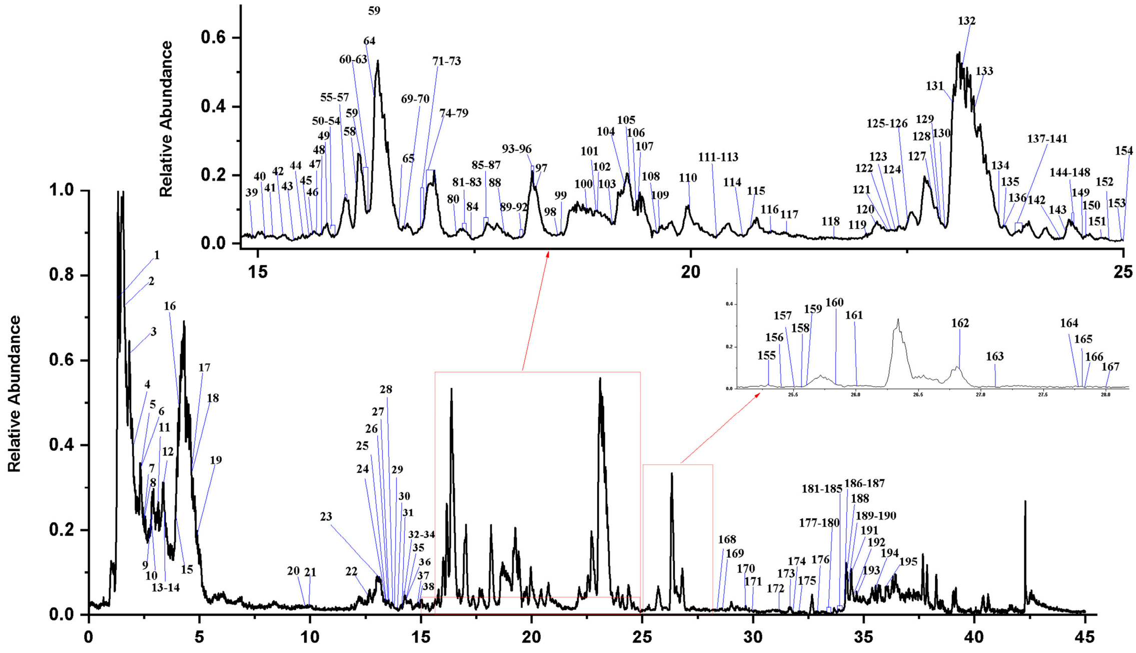

Figure S6. TIC of radish seed extracts from LC-MS analysis, with the major peaks annotated.

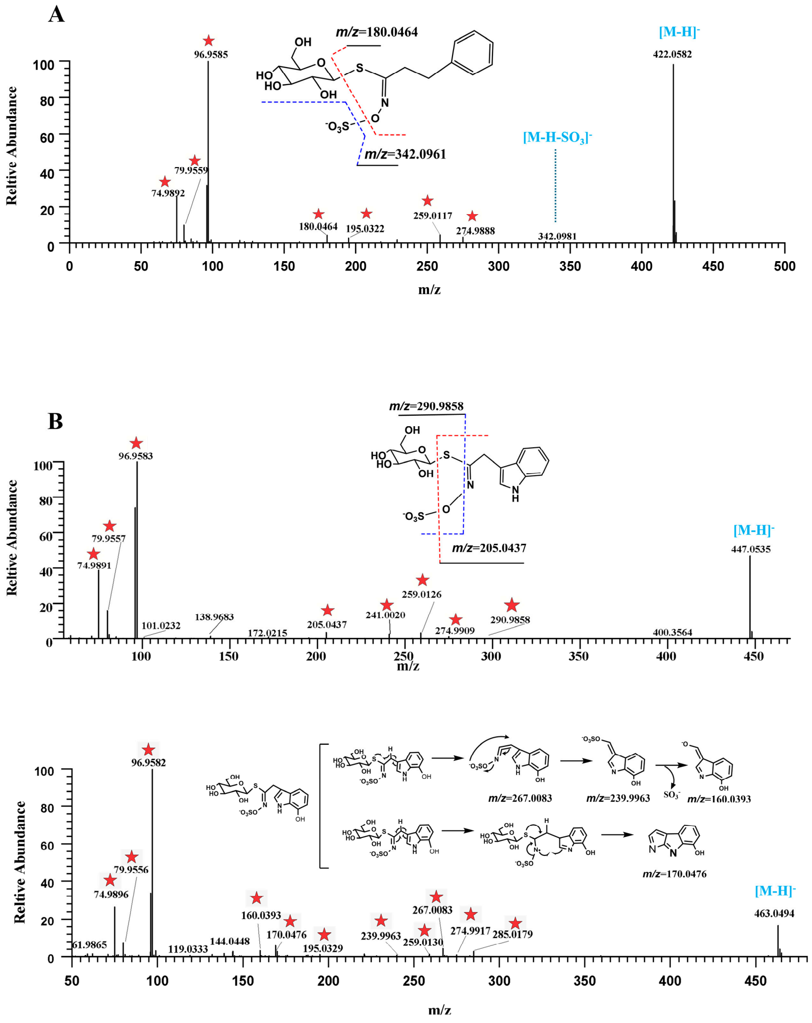
A
m/z=180.0464
m/z=342.0961
[M-H]-
[M-H-SO3]-
Reltive Abundance
m/z
96.9585
79.9559
74.9892
180.0464
195.0322
259.0117
274.9888
342.0981
422.0582
B
m/z=290.9858
m/z=205.0437
[M-H]-
96.9583
79.9557
74.9891
101.0232
138.9683
172.0215
205.0437
241.0020
259.0126
274.9909
290.9858
400.3564
447.0535
m/z=267.0083
m/z=239.9963
m/z=160.0393
m/z=170.0476
96.9582
79.9556
74.9896
61.9865
119.0333
144.0448
160.0393
170.0476
195.0329
239.9963
259.0130
267.0083
274.9917
285.0179
463.0494

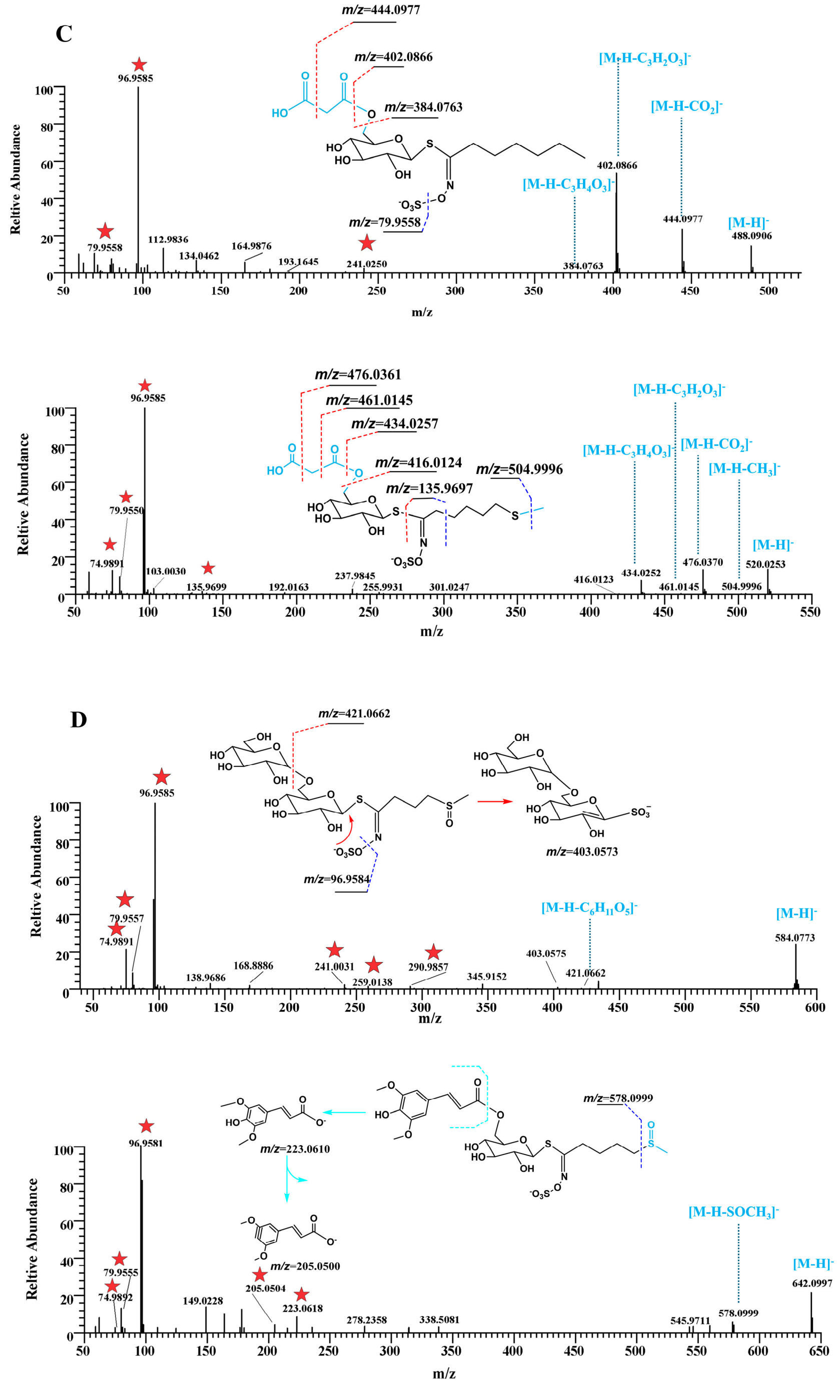

Figure S7. MS/MS spectra and fragmentation pathways of representative GSLs. (A) Compound 93; (B) Compounds 72 and 132; (C) Compounds 34 and 150; (D) Compounds 3 and 118.

Table S1. Comparison of network metrics between conventional FBMN and GSLsTracerMN.

Metric	Conventional FBMN	GSLsTracerMN
Total nodes	223	19
GSL-related nodes	14	12
Background/redundant nodes	209	7

Proportion of GSL-related nodes	6.28%	63.16%

Table S2. The chromatographic and mass spectral data of 195 compounds were characterized by UHPLC-Q-Exactive Orbitrap-MS.

NO	RT (min)	[M-H]- (m/z)	Error ppm	Formula	MS/MS fragment ions (m/z)	Side-chain identification	Class
1	1.44	604.1729	-0.7	$C_{22}H_{38}NO_{14}S_2$	259.0125, 241.0022, 96.9584, 79.9554, 74.9894	nonenyl or isomer	d
2	1.61	596.0776	-0.21	$C_{18}H_{30}NO_{15}S_3$	354.0680, 259.0122, 241.0018, 138.9692, 128.9312, 96.9584, 79.9556, 74.9893	12-(methylsulfinyl)dodecyl or isomer	d
3	1.8	584.0773	-0.78	$C_{17}H_{30}NO_{15}S_3$	421.0662, 290.9857, 259.0138, 241.0031, 96.9585, 79.9557, 74.9891	2-(methylsulfinyl)ethyl or isomer	d
4	1.99	566.0645	1.25	$C_{20}H_{24}NO_{14}S_2$	324.0579, 290.9856, 259.0123, 241.0019, 138.9694, 96.9584, 79.9557, 74.9893	monoly/1-(benzoyloxy)propanyl or isomer	b
5	2.34	452.0318	1.33	$C_{15}H_{18}NO_{11}S_2$	274.9896, 259.0121, 255.9966, 210.0262, 195.0323, 138.9690, 128.9304, 96.9584, 79.9556, 74.9893	(benzoyloxy)methyl or isomer	a
6	2.35	696.2031	0.27	$C_{25}H_{46}NO_{15}S_3$	259.0124, 241.0023, 96.9586, 79.9557, 74.9893	11-(methylsulfinyl)undecyl or isomer	d
7	2.49	422.0255	-0.03	$C_{11}H_{20}NO_{10}S_3$	407.0007, 358.0272, 274.9895, 259.0128, 241.0017, 195.0313, 164.0008, 96.9583, 79.9556, 74.9890	3-(methylsulfinyl)ethyl*	a
8	2.49	746.1242	-0.72	$C_{30}H_{36}NO_{15}S_3$	259.0129, 241.0023, 96.9584, 79.9556, 74.9894	Benzyl-sulfonyl-pentenyl# or isomer	c
9	2.83	566.0675	0.59	$C_{17}H_{28}NO_{14}S_3$	324.0579, 290.9856, 259.0123, 241.0009, 138.9694, 96.9584, 79.9557, 74.9893	3-(methylthio)allyl or isomer	d
10	2.87	500.1069	-2.65	$C_{18}H_{30}NO_9S_3$	304.0617, 259.0121, 241.0009, 96.9584, 79.9557, 74.9893	Butenyl-thio-heptene or isomer	a
11	3.16	388.0377	1.25	$C_{11}H_{18}NO_{10}S_2$	332.0111, 308.0795, 274.9905, 259.0127, 241.0023， 210.00,67, 195.0324, 96.9583, 79.9550, 74.9897	2-hydroxybutenyl*	a
12	3.31	463.9991	-0.11	$C_{12}H_{18}NO_{12}S_3$	274.9903, 259.0134, 241.0025, 96.9584, 79.9558, 74.9895	2-mercaptoethyl or isomer	b
13	3.44	602.054	3.46	$C_{16}H_{28}NO_{17}S_3$	259.0129, 138.9689, 135.9696, 96.9584, 79.9557, 74.9893	2-hydroxy-2-(methylsulfonyl)ethyl or isomer	d
14	3.47	776.2479	4.46	$C_{34}H_{50}NO_{13}S_3$	274.9903, 259.0134, 241.0021, 96.9584, 79.9557, 74.9892	12-((butenyl)thio)dodecenyl or isomer	c
15	3.87	440.0325	0.79	$C_{14}H_{18}NO_{11}S_2$	259.0135, 138.9691, 96.9584, 96.9496, 79.9556, 74.9893	3,4-dihydroxybenzyl or isomer	a
16	4	450.0184	-3.26	$C_{12}H_{20}NO_{11}S_3$	274.9916, 259.0133, 208.0088, 138.9688, 96.9584, 79.9558, 74.9893	4-(methylsulfonyl)butenyl or isomer	a
17	4.63	434.0249	-0.71	$C_{12}H_{20}NO_{10}S_3$	274.9893, 259.0122, 241.0008, 195.0325, 192.0153, 138.9688, 135.9692, 96.9584, 79.9556, 74.9893	12-(methylsulfinyl)dodecyl or isomer	a
18	4.67	436.0416	2.33	$C_{12}H_{22}NO_{10}S_3$	421.0183, 372.0410, 274.9927, 259.0131, 195.0325, 178.0172, 96.9590, 79.9556, 74.9895	4-(methylsulfinyl)butyl*	a
19	4.89	586.0591	3.61	$C_{16}H_{28}NO_{16}S_3$	259.0126, 241.0021, 138.9686, 96.9584, 79.9558, 74.9892	2-hydroxy-2-(methylsulfinyl)ethyl or isomer	d
20	9.77	372.0427	1.08	$C_{11}H_{18}NO_9S_2$	274.9902, 259.0128, 241.0016, 195.0319, 96.9583, 79.9556, 74.9896	butenyl*	a
21	9.91	450.0565	0.51	$C_{13}H_{24}NO_{10}S_3$	386,0586，370.1008，274.,9907, 259.0121, 192.0336, 138.9651, 96.9584, 79.9556, 74.9892	1-hydroxy-5-(methylthio)pentyl or isomer	a
22	12.58	448.0409	0.63	$C_{13}H_{22}NO_{10}S_3$	274.9902, 259.0127, 138.9685, 96.9584, 79.9556, 74.9893	5-Methylthio-oxopentyl or isomer	a
23	13.18	424.0353	-4.42	$C_{14}H_{18}NO_{10}S_2$	274.9902, 259.0127, 241.0012, 96.9588, 79.9560, 74.9895	2-hydroxybenzyl or isomer	a

24	13.41	550.0366	1.35	$C_{16}H_{24}NO_{14}S_3$	259.0131, 195.0323, 138.9787, 96.9584, 79.9558, 74.9892	5-(methylsulfinyl)-5-oxopentyl[#] or isomer	b
25	13.43	548.0951	3.76	$C_{18}H_{30}NO_{12}S_3$	259.0127, 195.0327, 96.9584, 79.9556, 74.9893	7-(methylthio)heptyl or isomer	b
26	13.5	476.0723	0.93	$C_{15}H_{26}NO_{10}S_3$	280.0331, 96.9585, 79.9557, 74.9893	7-Methylthio-3-oxoheptyl or isomer	a
27	13.6	418.0845	0.7	$C_{13}H_{24}NO_{10}S_2$	259.0113, 138.9686, 96.9584, 79.9556, 74.9893	6-hydroxyhexyl or isomer	a
28	13.72	406.0302	0.53	$C_{11}H_{20}NO_9S_3$	259.0117, 195.0324, 96.9584, 79.9556, 74.9893	3-(methylthio)propyl or isomer	a
29	13.78	614.0697	4.01	$C_{21}H_{28}NO_{14}S_3$	274.9903, 259.0131, 241.0023, 180.0409, 96.9584, 79.9558, 74.9892	2-(methylsulfinyl)ethyl or isomer	c
30	14.03	374.0583	0.84	$C_{11}H_{20}NO_9S_2$	274.9902, 259.0127, 241.0015, 96.9584, 79.9558, 74.9893	butyl or isomer	a
31	14.15	577.0777	-3.67	$C_{21}H_{25}N_2O_{13}S_2$	259.0127, 241.0013, 135.9690, 96.9584, 79.9555, 74.9893	2-(2-(3-hydroxy-2-oxoindolinyl)acetoxy)butenyl[#] or isomer	a
32	14.2	600.0554	0.88	$C_{17}H_{30}NO_{14}S_4$	274.9904, 259.0123, 241.0013, 96.9584, 79.9556, 74.9893	(glucosyldisulfanyl)Butyl or isomer	a
33	14.22	506.0461	0.06	$C_{15}H_{24}NO_{12}S_3$	259.0131, 135.9693, 96.9584, 79.9558, 74.9894	4-(methylthio)butyl or isomer	b
34	14.23	520.0253	-0.05	$C_{15}H_{22}NO_{13}S_3$	504.9996, 476.0370, 461.0145, 434.0252, 416.0124, 135.9699, 96.9585, 79.9550, 74.9891	12-(methylsulfinyl)dodecyl or isomer	b
35	14.31	388.0739	0.69	$C_{12}H_{22}NO_9S_2$	274.9903, 259.0125, 241.0014, 96.9583, 79.9558, 74.9892	pentanyl or isomer	a
36	14.37	628.1173	2.3	$C_{26}H_{30}NO_{13}S_2$	274.9903, 259.0131, 241.0023, 96.9584, 79.9558	phenethyl or isomer	c
37	14.56	548.1288	-1.02	$C_{19}H_{34}NO_{11}S_3$	274.9905, 259.0119, 241.0016, 96.9584, 74.9892	11-(methylsulfinyl)-11-oxoundecyl or isomer	a
38	14.83	466.0462	-3.46	$C_{16}H_{20}NO_{11}S_2$	274.9905, 259.0119, 241.0016, 96.9584, 79.9556, 74.9893	2-(benzoyloxy)ethyl or isomer	a
39	14.95	490.0879	0.73	$C_{16}H_{28}NO_{10}S_3$	294.0487, 96.9585, 74.9893	8-Methylthio-3-oxooctyl or isomer	a
40	15.08	458.0617	0.76	$C_{15}H_{24}NO_9S_3$	259.0127, 241.0012, 96.9585, 79.9558, 74.9894	4-((butenyl)thio)butenyl or isomer	a
41	15.17	550.0729	1.14	$C_{17}H_{28}NO_{13}S_3$	259.0135, 135.9695, 96.9584, 79.9557, 74.9891	1-hydroxy-6-(methylthio)hexyl[#] or isomer	b
42	15.27	548.0574	1.33	$C_{17}H_{26}NO_{13}S_3$	259.0131, 241.0028, 135.9699, 96.9584, 79.9557, 74.9894	6-Methylthio-oxohexyl or isomer	b
43	15.4	625.1013	0.65	$C_{22}H_{29}N_2O_{15}S_2$	290.9843, 259.0127, 241.0021, 138.9692, 96.9584, 79.9557, 74.9893	(4-hydroxy-1H-indolyl)methyl[#] or isomer	d
44	15.51	386.0584	1.12	$C_{12}H_{20}NO_9S_2$	195.0321, 190.0192, 144.0488, 96.9584, 79.9556, 74.9893	3-methylbutenyl or isomer	a
45	15.59	500.0881	-3.08	$C_{17}H_{26}NO_{12}S_2$	259.0134, 241.0028, 195.0321, 96.9584, 74.9892	hepenyl or isomer	b
46	15.62	514.1245	1.06	$C_{19}H_{32}NO_9S_3$	352.0637, 259.0125, 241.0014, 96.9585, 74.9896	8-((butenyl)thio)octenyl or isomer	a
47	15.65	404.0668	-4.15	$C_{12}H_{22}NO_{10}S_2$	241.0016, 180.0437, 138.9687, 96.9584, 79.9558, 74.9893	2-hydroxymethylbutyl or isomer	a
48	15.73	534.0413	0.6	$C_{16}H_{24}NO_{13}S_3$	241.0026, 195.0319, 135.9694, 96.9584, 79.9557, 74.9893	5-Methylthio-oxopentyl or isomer	b
49	15.79	520.0615	-0.34	$C_{16}H_{26}NO_{12}S_3$	259.0134, 135.9697, 96.9584, 79.9591, 74.9893	2-ethylbutyl or isomer	b
50	15.81	390.053	0.34	$C_{11}H_{20}NO_{10}S_2$	241.0018, 148.0409, 96.9584, 79.9558, 74.9894	2-(hydroxymethyl)butyl or isomer	a
51	15.83	578.0677	0.85	$C_{18}H_{28}NO_{14}S_3$	259.0134, 195.0324, 135.9694, 96.9584, 79.9556, 74.9892	7-(methylsulfinyl)-7-oxoheptyl[#] or isomer	b
52	15.84	464.0358	0.57	$C_{13}H_{22}NO_{11}S_3$	259.0124, 241.0015, 138.9694, 96.9584, 79.9556, 74.9893	5-(methylsulfinyl)-5-oxopentyl or isomer	a
53	15.87	567.0814	2.06	$C_{16}H_{27}N_2O_{16}S_2$	389.0520, 259.0127, 96.9583, 79.9556, 74.9893	3-amino-3-carboxypropyl or isomer	d
54	15.87	550.0914	2.5	$C_{17}H_{28}NO_{15}S_2$	274.9901, 259.0124, 195.0319, 180.0477, 96.9584, 79.9558, 74.9893	2-hydroxybutenyl or isomer	d
55	16	839.0846	1.3	$C_{24}H_{43}N_2O_{18}S_6$	274.9934, 259.0131, 138.9685, 96.9584, 79.9557, 74.9893	Dimeric 4-mercaptoHexyl or isomer	a
56	16.02	408.0426	0.68	$C_{14}H_{18}NO_9S_2$	274.9891, 259.0129, 195.0328, 166.0330, 138.9699, 96.9584, 79.9556, 74.9893	benzyl[*]	a
57	16.03	715.1157	-3.61	$C_{22}H_{39}N_2O_{16}S_4$	274.9903, 259.0128, 96.9584, 79.9557, 74.9895	6-((2-amino-2-carboxyethyl)disulfaneyl)hexyl[#] or isomer	d

58	16.13	482.0463	0.47	$C_{13}H_{24}NO_{12}S_3$	259.0124, 195.0318, 96.9584, 74.9893	5-hydroxy-5-(methylsulfonyl)pentyl sulfate or isomer	a
59	16.17	358.0272	1.52	$C_{10}H_{16}NO_9S_2$	278.0666, 241.0017, 96.9584, 79.9568, 74.9893	allyl or isomer	a
60	16.24	418.0294	-1.51	$C_{12}H_{20}NO_9S_3$	290.9853, 274.9901, 259.0124, 241.0014, 227.0222, 195.0324, 176.0198, 138.9691, 96.9584, 79.9556, 74.9893	4-(methylthio)butenyl or isomer	a
61	16.25	420.0459	0.62	$C_{12}H_{22}NO_9S_3$	340.0891, 290.9852, 274.9900, 259.0130, 241.0014, 227.0224, 195.0321, 138.9692, 96.9584, 79.9568, 74.9893	4-(methylthio)butyl*	a
62	16.29	671.0891	0.62	$C_{23}H_{31}N_2O_{15}S_3$	274.9905, 259.0130, 241.0023, 195.0324, 96.9584, 79.9557, 74.9893	2-((2-amino-2-carboxyethyl)thio)ethyl# or isomer	c
63	16.34	534.0771	-0.4	$C_{17}H_{28}NO_{12}S_3$	259.0134, 138.9687, 135.9695, 96.9584, 79.9556, 74.9893	6-(methylthio)hexyl or isomer	b
64	16.51	532.062	0.45	$C_{17}H_{26}NO_{12}S_3$	241.0012, 195.0318, 138.9687, 135.9694, 128.9304, 96.9584, 79.9556, 74.9893	6-(methylthio)hexenyl or isomer	b
65	16.62	530.1347	-3.63	$C_{19}H_{32}NO_{12}S_2$	334.1055, 241.0028, 195.0323, 96.9584, 79.9560, 74.9893	nonyl or isomer	b
66	16.69	598.0729	0.98	$C_{21}H_{28}NO_{13}S_3$	259.0123, 241.0019, 96.9584, 79.9557, 74.9893	2-(methylthio)ethyl or isomer	c
67	16.71	628.0494	4.64	$C_{21}H_{26}NO_{15}S_3$	274.9905, 259.0130, 241.0023, 96.9584, 79.9558, 74.9894	2-(methylsulfinyl)-2-oxoethyl# or isomer	c
68	16.76	514.1062	1.74	$C_{18}H_{28}NO_{12}S_2$	272.0896, 259.0135, 96.9585, 79.9558, 74.9896	octenyl or isomer	b
69	16.77	507.0745	0.24	$C_{18}H_{23}N_2O_{11}S_2$	329.0451, 311.0353, 195.0317, 138.9691, 96.9584, 79.9560, 74.9892	(1,4-dimethoxyindolyl)methyl or isomer	a
70	16.77	448.0231	2.6	$C_{12}H_{18}NO_{13}S_2$	274.9902, 259.0134, 195.0321, 96.9585, 79.9558, 74.9892	2-hydroxyethyl or isomer	b
71	16.86	609.1064	0.59	$C_{22}H_{29}N_2O_{14}S_2$	274.9901, 259.0124, 96.9584, 79.9557, 74.9893	(1H-indolyl)methyl or isomer	d
72	16.89	447.0535	0.68	$C_{16}H_{19}N_2O_9S_2$	290.9858, 274.9909, 259.0126, 241.0020, 205.0437, 96.9583, 79.9557, 74.9891	(indolyl)methyl*	a
73	16.89	628.0831	0.38	$C_{22}H_{30}NO_{14}S_3$	274.9901, 259.0125, 135.9696, 96.9584, 79.9557, 74.9892	2-(methylsulfinyl)ethyl or isomer	c
74	16.93	742.1861	-1.69	$C_{29}H_{44}NO_{15}S_3$	274.9888, 259.0129, 205.0424, 195.0317, 138.9696, 128.9307, 96.9584, 79.9556, 74.9858	10-(methylsulfonyl)decyl or isomer	c
75	16.96	527.01	-0.06	$C_{16}H_{19}N_2O_{12}S_3$	447.0532, 274.9894, 259.0128, 241.0016, 138.9692, 96.9584, 79.9568, 74.9894	(1-sulfoindolyl)methyl or isomer	a
76	16.96	723.2108	0.46	$C_{29}H_{43}N_2O_{15}S_2$	274.9899, 259.0128, 205.0428, 195.0321, 138.9689, 128.9306, 96.9584, 79.9556, 74.9893	11-amino-11-carboxyundecyl# or isomer	c
77	16.98	658.0916	2.39	$C_{26}H_{28}NO_{15}S_2$	274.9905, 259.0130, 241.0023, 223.0601, 135.9693, 128.9307, 96.9584, 79.9557, 74.9893	(benzoyloxy)methyl or isomer	c
78	17.11	580.0807	2.07	$C_{21}H_{26}NO_{14}S_2$	274.9902, 241.0025, 96.9584, 79.9556, 74.9894	4-(benzoyloxy)butyl or isomer	b
79	17.11	580.0827	-0.29	$C_{18}H_{30}N_1O_{14}S_3$	259.0124, 241.0025, 96.9584, 79.9556, 74.9894	4-(methylthio)butenyl or isomer	d
80	17.29	626.0677	0.87	$C_{22}H_{28}NO_{14}S_3$	274.9902, 259.0134, 96.9584, 79.9557, 74.9894	Benzyl-sulfonyl-pentenyl# or isomer	b
81	17.37	570.0413	0.49	$C_{19}H_{24}NO_{13}S_3$	259.0128, 195.0301, 135.9701, 96.9584, 79.9557, 74.9896	mercaptomethyl or isomer	c
82	17.39	492.1037	1	$C_{16}H_{30}NO_{10}S_3$	290.9851, 274.9880, 259.0132, 250.0941, 241.0013, 195.0320, 138.9691, 135.9690, 128.9305, 96.9584, 79.9556, 74.9893	8-(methylsulfinyl)octyl or isomer	a
83	17.4	432.0468	2.68	$C_{13}H_{22}NO_9S_3$	274.9894, 259.0128, 241.0016, 96.9585, 79.9555, 74.9893	5-(methylthio)pentenyl or isomer	a
84	17.45	460.0775	1.2	$C_{15}H_{26}NO_9S_3$	264.0383, 195.0324, 96.9585, 79.9555, 74.9893	7-Methylthio-6-heptenyl or isomer	a
85	17.6	553.0625	-3.72	$C_{15}H_{25}N_2O_{16}S_2$	259.0123, 241.0021, 135.9694, 128.9295, 96.9584, 79.9557, 74.9892	2-amino-2-carboxyethyl or isomer	d
86	17.62	462.0566	0.91	$C_{14}H_{24}NO_{10}S_3$	274.9894, 259.0128, 241.0019, 195.0323, 96.9585, 74.9892	6-Methylthio-oxohexyl or isomer	a
87	17.63	488.1086	0.76	$C_{17}H_{30}NO_9S_3$	292.0694, 241.0015, 96.9584, 79.9559, 74.9893	9-(methylthio)nonenyl or isomer	a

88	17.84	493.0611	-1.92	$C_{14}H_{25}N_2O_{11}S_3$	259.0126, 241.0031, 96.9584, 79.9557, 74.9894	(Cystein-S-yl)butyl or isomer	a
89	18.04	550.1426	1.6	$C_{22}H_{32}NO_{11}S_2$	274.9901, 259.0128, 96.9584, 79.9558, 74.9895	8-(benzoyloxy)octyl or isomer	a
90	18.04	550.1256	-1.48	$C_{18}H_{32}NO_{14}S_2$	259.0126, 241.0025, 96.9584, 79.9558, 74.9895	pentanyl or isomer	d
91	18.09	577.1558	-0.25	$C_{20}H_{37}N_2O_{11}S_3$	259.0127, 241.0028, 96.9584, 79.9557, 74.9893	(Cystein-S-yl)decyll# or isomer	a
92	18.09	400.0737	0.28	$C_{13}H_{22}NO_9S_2$	274.9901, 259.0127, 96.9584, 79.9556, 74.9893	hexenyl or isomer	a
93	18.1	422.0582	0.57	$C_{15}H_{20}NO_9S_2$	342.0961,274.9888，259.0117,195.0322，180.0464, 96.9585, 79.9559, 74.9892	phenylethyl*	a
94	18.12	726.1904	-2.78	$C_{29}H_{44}NO_{14}S_3$	274.9906, 259.0128, 205.0495, 180.0474, 138.9701, 96.9584, 79.9557, 74.9856	10-(methylsulfinyl)decyl# or isomer	c
95	18.12	845.1188	-1.11	$C_{25}H_{41}N_4O_{20}S_4$	290.9892, 274.9909, 259.0124, 241.0019, 195.0321, 180.0476, 138.9691, 128.9303, 96.9584, 79.9556, 74.9893	Glutathione disulfanyl ethyl# or isomer	d
96	18.13	867.1152	0.5	$C_{26}H_{47}N_2O_{18}S_6$	259.0126, 195.0304, 180.0472, 138.9690, 96.9584, 79.9556, 74.9893	Dimeric 4-mercaptoHexyl or isomer	a
97	18.18	434.0609	-0.94	$C_{13}H_{24}NO_9S_3$	274.9900, 259.0133, 241.0014, 192.0513, 138.9688, 128.9307, 96.9584, 79.9556, 74.9893	2-ethylbutyl or isomer	a
98	18.46	474.0928	0.37	$C_{16}H_{28}NO_9S_3$	278.0536, 96.9585, 74.9893	8-(methylthio)octenyl or isomer	a
99	18.51	516.1206	-0.73	$C_{18}H_{30}NO_{12}S_2$	354.0644, 274.9902, 259.0134, 96.9582, 74.9894	octyl or isomer	b
100	18.79	576.0886	1.15	$C_{19}H_{30}NO_{13}S_3$	274.9901, 259.0131, 241.0028, 135.9694, 96.9584, 79.9556	8-Methylthio-oxooctyl or isomer	b
101	18.94	402.0541	2.98	$C_{12}H_{20}NO_{10}S_2$	274.9901, 259.0124, 241.0014, 239.9967, 96.9587, 74.9894	2-hydroxypentenyl or isomer	a
102	18.95	640.083	0.29	$C_{23}H_{30}NO_{14}S_3$	274.9899, 259.0134, 241.0026, 135.9696, 96.9585, 79.9556, 74.9892	6-(benzylthio)hexenyl or isomer	b
103	19.08	584.057	0.69	$C_{20}H_{26}NO_{13}S_3$	259.0123, 138.9680, 135.9692, 128.9302, 96.9584, 79.9556	2-mercaptoethyl or isomer	c
104	19.24	783.1411	4.26	$C_{32}H_{35}N_2O_{17}S_2$	259.0120, 241.0013, 138.9691, 96.9584, 79.9556, 74.9893	2-(2-(3-hydroxy-2-oxoindolinyl)acetoxy)butenyl# or isomer	c
105	19.29	534.096	1.66	$C_{17}H_{28}NO_{14}S_2$	259.0125, 241.0023, 195.0323, 96.9584, 79.9557, 74.9892	butenyl or isomer	d
106	19.36	614.0707	0.15	$C_{18}H_{32}NO_{14}S_4$	274.9903, 259.0121, 241.0013, 180.0409, 135.9694, 96.9584, 79.9556, 74.9892	(glucosyldisulfanyl)Pentyl or isomer	a
107	19.39	504.0316	2.42	$C_{15}H_{22}NO_{12}S_3$	274.9899, 241.0026, 96.9584, 79.9558, 74.9894	4-(methylthio)butenyl or isomer	b
108	19.61	614.1016	2.27	$C_{25}H_{28}NO_{13}S_2$	259.0124, 223.0606, 205.0497, 190.0263, 138.9689, 135.9693, 128.9306, 96.9584, 79.9556, 74.9892	benzyl or isomer	c
109	19.64	517.1546	3.81	$C_{18}H_{33}N_2O_{11}S_2$	355.1028, 290.9856, 274.9903, 259.0123, 241.0015, 96.9585, 74.9893	11-amino-11-carboxyundecyl or isomer	a
110	19.98	402.0893	0.07	$C_{13}H_{24}NO_9S_2$	290.9856, 274.9903, 259.0121, 241.0010, 195.0334, 96.9584, 79.9557, 74.9858	hexyl or isomer	a
111	20.3	502.1243	0.74	$C_{18}H_{32}NO_9S_3$	306.0851, 241.0008, 96.9584, 74.9892	10-(methylthio)decenyl or isomer	a
112	20.36	465.032	2.67	$C_{12}H_{21}N_2O_{11}S_3$	259.0121, 241.0017, 138.9692, 96.9584, 79.9557, 74.9896	(Cystein-S-yl)ethyl# or isomer	a
113	20.37	612.0879	-0.09	$C_{22}H_{30}NO_{13}S_3$	274.9905, 259.0130, 241.0023, 135.9697, 96.9584, 79.9559	3-(methylthio)propyl or isomer	c
114	20.59	520.0977	-0.78	$C_{17}H_{30}NO_{11}S_3$	274.9904, 259.0124, 241.0016, 96.9584, 79.9558, 74.9892	9-(methylsulfinyl)-9-oxononyl or isomer	a
115	20.69	672.1063	0.85	$C_{27}H_{30}NO_{15}S_2$	259.0130, 135.9693, 96.9584, 79.9555, 74.9894	2-(benzoyloxy)ethyl or isomer	c
116	20.91	553.0643	-2.04	$C_{16}H_{29}N_2O_{11}S_4$	135.9704, 128.9317, 96.9584, 79.9556, 74.9896	6-((2-amino-2-carboxyethyl)disulfaneyl)hexyl# or isomer	a
117	21.1	595.1146	3.76	$C_{19}H_{35}N_2O_{11}S_4$	399.0764, 96.9585, 79.9558, 74.9893	9-((2-amino-2-carboxyethyl)disulfaneyl)nonyl# or isomer	a

118	21.65	642.0997	1.87	$C_{23}H_{32}NO_{14}S_3$	578.0999, 223.0618, 205.0504, 96.9581, 79.9555, 74.9892	2-(methylsulfinyl)butyl or isomer	c
119	21.94	405.995	3.35	$C_{10}H_{16}NO_{10}S_3$	274.9884, 259.0125, 96.9585, 79.9557	2-Methylthio-oxoethyl or isomer	a
120	22.13	670.0941	0.96	$C_{24}H_{32}NO_{15}S_3$	290.9858, 259.0117, 138.9686, 96.9584, 79.9557, 74.9892	5-(methylsulfinyl)-5-oxopentyl# or isomer	c
121	22.28	444.0465	1.86	$C_{14}H_{22}NO_9S_3$	259.0135, 241.0011, 96.9587, 79.9586	3-((buten-yl)thio)allyl or isomer	a
122	22.3	414.0901	2.17	$C_{14}H_{24}NO_9S_2$	259.0134, 241.0013, 96.9584, 79.9558, 74.9893	heptenyl or isomer	a
123	22.36	540.0671	0.5	$C_{19}H_{26}NO_{11}S_3$	259.0134, 241.0013, 96.9584, 79.9557, 74.9893	Benzyl-sulfonyl-pentenyl or isomer	a
124	22.41	536.1088	-3.75	$C_{17}H_{30}NO_{14}S_2$	340.0696, 274.9901, 259.0124, 96.9585, 79.9558, 74.9893	butyl or isomer	d
125	22.48	742.1152	-3.64	$C_{24}H_{40}NO_{17}S_4$	259.0134, 96.9587, 79.9556, 74.9894	(glucosyldisulfanyl)octyl or isomer	b
126	22.49	656.0784	0.92	$C_{23}H_{30}NO_{15}S_3$	259.0127, 223.0603, 205.0499, 138.9701, 135.9693, 128.9306, 96.9584, 79.9556, 74.9897	4-(methylsulfonyl)butenyl or isomer	c
127	22.76	642.1302	-2.03	$C_{27}H_{32}NO_{13}S_2$	446.0910, 259.0130, 223.0603, 190.0262, 205.0493, 138.9699, 135.9692, 96.9584, 79.9556, 74.9892	3-phenylpropyl or isomer	c
128	22.87	556.0795	0.03	$C_{19}H_{26}N_1O_{14}S_2$	274.9905, 259.0125, 241.0023, 96.9584, 79.9556, 74.9891	phenyl or isomer	d
129	22.88	610.0723	0.1	$C_{22}H_{28}NO_{13}S_3$	259.0130, 227.0246, 223.0601, 138.9689, 135.9694, 96.9584, 79.9557	3-(methylthio)allyl or isomer	c
130	22.93	595.1136	3.55	$C_{18}H_{31}N_2O_{16}S_2$	399.0764, 259.0121, 96.9585, 79.9558, 74.9893	5-amino-5-carboxypentyl or isomer	d
131	23.05	497.003	0.32	$C_{12}H_{21}N_2O_{11}S_4$	417.0492, 241.0029, 138.9691, 96.9584, 79.9568	2-((2-amino-2-carboxyethyl)disulfaneyl)ethyl# or isomer	a
132	23.1	463.0494	2.78	$C_{16}H_{19}N_2O_{10}S_2$	285.0179, 274.9917, 267.0083, 259.0130, 239.9963, 195.0329, 170.0476, 160.0393, 96.9582, 79.9556, 74.9896	1-hydroxy-6-(methylthio)hexyl or isomer	a
133	23.27	490.0689	0.06	$C_{15}H_{24}NO_{13}S_2$	294.0297, 241.0023, 96.9584, 79.9557, 74.9893	2-hydroxy-2-methylbutyl# or isomer	b
134	23.54	656.1361	1.26	$C_{21}H_{38}NO_{16}S_3$	274.9903, 259.0124, 241.0021, 96.9584, 79.9555, 74.9892	7-(methylsulfonyl)heptyl or isomer	d
135	23.61	563.1401	-0.28	$C_{19}H_{35}N_2O_{11}S_3$	274.9884, 259.0125, 96.9584, 79.9557, 74.9892	(Cystein-S-yl)nonyl or isomer	a
136	23.62	626.1263	2.53	$C_{20}H_{36}NO_{15}S_3$	430.0971, 274.9901, 259.0121, 96.9584, 79.9556, 74.9891	1-hydroxy-6-(methylthio)hexyl or isomer	d
137	23.74	628.0853	-1.42	$C_{19}H_{34}NO_{14}S_4$	274.9884, 259.0125, 135.9688, 96.9584, 79.9556, 74.9894	(glucosyldisulfanyl)Hexyl or isomer	a
138	23.75	656.1168	-1.07	$C_{21}H_{38}NO_{14}S_4$	96.9584, 79.9556, 74.9893	(glucosyldisulfanyl)Octyl or isomer	a
139	23.81	596.1303	1.58	$C_{23}H_{34}NO_{11}S_3$	274.9883, 259.0126, 96.9584, 74.9894	(E)-9-(benzylthio)non-8-en-1-yl or isomer	a
140	23.81	686.1575	-0.32	$C_{29}H_{36}NO_{14}S_2$	259.0120, 241.0018, 96.9585, 79.9555, 74.9890	2-(4-methoxyphenyl)-2-methylpropyl# or isomer	c
141	24.15	656.1461	-1.62	$C_{28}H_{34}NO_{13}S_2$	290.9858, 259.0119, 223.0605, 205.0503, 96.9584, 79.9555	4-phenylbutyl or isomer	c
142	24.27	658.1285	3.14	$C_{27}H_{32}NO_{14}S_2$	135.9691, 128.9296, 96.9584, 79.9556, 74.9893	4-methoxyphenethyl or isomer	c
143	24.33	627.0849	2.2	$C_{18}H_{31}N_2O_{16}S_3$	259.0125, 241.0019, 195.0323, 96.9584, 79.9557, 74.9893	2-((2-amino-2-carboxyethyl)thio)ethyl# or isomer	d
144	24.39	608.1114	-4.52	$C_{20}H_{34}NO_{14}S_3$	412.0622, 366.0918, 138.9694, 96.9584, 79.9557, 74.9893	9-(methylsulfonyl)nonyl or isomer	b
145	24.4	739.1306	-2.76	$C_{27}H_{35}N_2O_{18}S_2$	259.0124, 241.0018, 96.9584, 79.9557, 74.9893	2-(2-(3-hydroxy-2-oxoindolinyl)acetoxy)butenyl# or isomer	d
146	24.41	416.1052	0.81	$C_{14}H_{26}NO_9S_2$	274.9902, 259.0128, 174.0956, 138.9695, 96.9584, 79.9556, 74.9893	Heptyl or isomer	a
147	24.42	656.1147	0.82	$C_{24}H_{34}NO_{14}S_3$	274.9905, 259.0130, 223.0606, 205.0493, 190.0258, 96.9584, 79.9556, 74.9893	1-hydroxy-5-(methylthio)pentyl or isomer	c
148	24.42	649.1415	1.26	$C_{22}H_{37}N_2O_{14}S_3$	274.9901, 259.0131, 241.0028, 96.9585, 79.9562	9-((2-amino-2-carboxyethyl)thio)nonyl# or isomer	b
149	24.53	739.2092	0.54	$C_{26}H_{47}N_2O_{16}S_3$	259.0123, 241.0014, 195.0269, 96.9581, 74.9894	10-((2-amino-2-carboxyethyl)thio)decyl or isomer	d

150	24.56	488.0906	1.97	$C_{16}H_{26}NO_{12}S_2$	444.0977, 402.0866, 384.0763, 241.0250, 96.9585, 79.9558	hexyl or isomer	b
151	24.74	640.1417	2.05	$C_{21}H_{38}NO_{15}S_3$	259.0126, 135.9697, 96.9584, 79.9556, 74.9893	7-(methylsulfinyl)heptyl or isomer	d
152	24.83	535.109	-0.07	$C_{17}H_{31}N_2O_{11}S_3$	290.9856, 274.9902, 259.0128, 96.9581, 79.9557, 74.9896	(Cystein-S-yl)heptyl or isomer	a
153	24.99	624.1618	1.82	$C_{25}H_{38}NO_{11}S_3$	138.9682, 135.9694, 96.9584, 79.9559, 74.9894	(E)-11-(benzylthio)undec-10-en-1-yl or isomer	a
154	25	618.1364	2.4	$C_{22}H_{36}NO_{13}S_3$	259.0134, 135.9693, 96.9584, 79.9557, 74.9892	11-(methylsulfinyl)undec-10-enyl# or isomer	b
155	25.33	608.1119	1.83	$C_{23}H_{30}NO_{14}S_2$	412.0622, 366.0918, 138.9694, 96.9584, 79.9557, 74.9893	2-hydroxypentenyl or isomer	c
156	25.38	686.1475	2.42	$C_{22}H_{40}NO_{17}S_3$	259.0124, 241.0019, 195.0323, 96.9585, 79.9555, 74.9890	8-hydroxy-8-(methylsulfonyl)octyl or isomer	d
157	25.5	563.0634	-1.3	$C_{20}H_{23}N_2O_{13}S_2$	274.9903, 259.0134, 96.9584, 79.9556, 74.9895	(1-methoxy-1H-indolyl)methyl# or isomer	b
158	25.57	787.1514	-4.08	$C_{29}H_{43}N_2O_{15}S_4$	274.9905, 259.0126, 205.0501, 190.0262, 96.9584, 79.9557, 74.9892	8-((2-amino-2-carboxyethyl)disulfaneyl)octyl# or isomer	c
159	25.6	710.2	0.35	$C_{23}H_{30}NO_{13}S_3$	259.0125, 241.0019, 96.9584, 79.9557, 74.9894	4-(methylthio)butenyl# or isomer	c
160	25.83	669.1064	0.64	$C_{27}H_{29}N_2O_{14}S_2$	290.9854, 259.0123, 223.0601, 96.9584, 79.9557, 74.9895	(4-hydroxy-1H-indolyl)methyl or isomer	c
161	25.95	680.1506	0.17	$C_{27}H_{38}NO_{13}S_3$	259.0124, 241.0019, 135.9695, 96.9584, 79.9556, 74.9893	8-(methylthio)octenyl or isomer	c
162	26.85	654.0991	0.91	$C_{24}H_{32}NO_{14}S_3$	259.0125, 241.0022, 135.9697, 96.9584, 79.9556, 74.9893	5-Methylthio-oxopentyl# or isomer	c
163	27.11	684.1459	-4.28	$C_{23}H_{42}NO_{14}S_4$	138.9684, 96.9584, 79.9560, 74.9895	(glucosyldisulfanyl)Decyl or isomer	a
164	27.77	640.1196	0.61	$C_{24}H_{34}NO_{13}S_3$	241.0019, 223.0606, 135.9697, 96.9584, 79.9556, 74.9894	2-ethylbutyl or isomer	c
165	27.81	447.0758	3.4	$C_{13}H_{23}N_2O_{11}S_2$	274.9903, 259.0124, 180.0421, 96.9588, 74.9893	6-amino-6-carboxyhexyl or isomer	a
166	27.83	510.0763	-2.16	$C_{15}H_{28}NO_{12}S_3$	274.9899, 259.0128, 195.0334, 96.9584, 79.9557	7-hydroxy-7-(methylsulfonyl)heptyl or isomer	a
167	27.97	546.1495	-1.22	$C_{20}H_{36}NO_{10}S_3$	274.9903, 259.0126, 195.0334, 96.9584, 79.9557	12-(methylsulfinyl)dodec-11-en-1-yl# or isomer	a
168	28.42	626.1042	0.94	$C_{23}H_{32}NO_{13}S_3$	274.9904, 259.0125, 223.0603, 205.0502, 96.9584, 79.9556, 74.9896	4-(methylthio)butyl# or isomer	c
169	28.54	642.1182	-2.2	$C_{20}H_{36}NO_{16}S_3$	446.0910, 259.0124, 241.0021, 138.9699, 135.9692, 96.9584, 79.9556, 74.9892	6-hydroxy-6-(methylsulfinyl)hexyl# or isomer	d
170	29.77	444.0966	-7.2	$C_{15}H_{26}NO_{10}S_2$	274.9902, 259.0123, 241.0037, 96.9584, 79.9558	5-oxooctyl or isomer	a
171	29.94	592.1163	0.78	$C_{23}H_{30}NO_{13}S_2$	259.0125, 223.0600, 227.0217, 205.0489, 138.9691, 96.9584, 79.9557, 74.9894	3-methylbutenyl or isomer	c
172	31.22	606.1318	0.42	$C_{24}H_{32}NO_{13}S_2$	274.9903, 259.0128, 223.0623, 96.9584, 79.9557, 74.9896	hexenyl or isomer	c
173	31.7	642.1519	-1.17	$C_{24}H_{36}NO_{15}S_2$	290.9893, 274.9905, 259.0124, 241.0013, 96.9585, 79.9559, 74.9892	2-(4-methoxyphenyl)-2-methylpropyl# or isomer	d
174	31.8	524.0723	-3.98	$C_{15}H_{26}NO_{15}S_2$	259.0123, 241.0018, 96.9584, 79.9557, 74.9894	2-hydroxyethyl or isomer	d
175	31.95	480.0637	0.49	$C_{17}H_{22}NO_{11}S_2$	259.0123, 241.0037, 195.0323, 96.9583, 74.9893	1-(benzoyloxy)propan-2-yl or isomer	a
176	32.94	608.1479	1.25	$C_{24}H_{34}NO_{13}S_2$	259.0126, 241.0020, 223.0610, 96.9584, 79.9560, 74.9893	hexyl or isomer	c
177	33.39	522.0927	-4.56	$C_{16}H_{28}NO_{14}S_2$	274.9905, 259.0121, 96.9585, 79.9554, 74.9894	isopropyl or isomer	d
178	33.45	655.113	-2.89	$C_{20}H_{35}N_2O_{16}S_3$	274.9903, 259.0125, 241.0019, 195.0323, 96.9583, 79.9559	4-((2-amino-2-carboxyethyl)thio)butyl# or isomer	d
179	33.48	610.0914	-3.23	$C_{19}H_{32}N_1O_{15}S_3$	259.0123, 241.0018, 195.0323, 96.9586, 74.9894	5-Methylthio-oxopentyl or isomer	d
180	33.81	474.0731	-1.89	$C_{15}H_{24}NO_{12}S_2$	259.0134, 241.0019, 96.9584, 79.9556, 74.9893	pentanyl or isomer	b
181	33.82	600.1253	1.59	$C_{22}H_{34}NO_{12}S_3$	259.0134, 241.0021, 96.9584, 79.9556, 74.9892	8-((butenyl)thio)octenyl or isomer	b
182	33.94	433.0592	1.16	$C_{12}H_{21}N_2O_{11}S_2$	290.9856, 274.9902, 259.0123, 180.0395, 96.9586, 79.9556	5-aminocarboxypentyl or isomer	a
183	33.95	479.0482	3.8	$C_{13}H_{23}N_2O_{11}S_3$	241.0014, 138.9688, 96.9584, 79.9557, 74.9895	Cystein-S-yl)propyl or isomer	a

184	34.02	636.1428	1.21	$C_{25}H_{34}NO_{14}S_2$	259.0124, 223.0603, 205.0501, 241.0023, 96.9584, 79.9555, 74.9893	4-oxoheptyl or isomer	c
185	34.05	512.0359	0.73	$C_{17}H_{22}NO_{11}S_3$	274.9902, 241.0016, 96.9584, 79.9558, 74.9893	3-(benzylthio)allyl or isomer	a
186	34.13	405.027	-0.94	$C_{10}H_{17}N_2O_{11}S_2$	274.9902, 259.0128, 241.0017, 96.9583, 79.9558, 74.9891	3-aminocarboxypropyl or isomer	a
187	34.16	472.0778	1.74	$C_{16}H_{26}NO_9S_3$	310.0250, 259.0127, 241.0017, 96.9584, 79.9556	Butenyl-thio-pentene or isomer	a
188	34.23	419.0441	2.66	$C_{11}H_{19}N_2O_{11}S_2$	274.9903, 259.0128, 241.0017, 96.9584, 79.9555	4-aminocarboxybutyl or isomer	a
189	34.34	532.1357	2.24	$C_{19}H_{34}NO_{10}S_3$	274.9903, 259.0126, 241.0017, 96.9580, 79.9559	11-(methylsulfinyl)undecenyl or isomer	a
190	34.37	592.0812	2.95	$C_{22}H_{26}NO_{14}S_2$	274.9903, 241.0023, 96.9584, 79.9557, 74.9892	2-(benzoyloxy)butenyl or isomer	b
191	34.42	704.133	1.59	$C_{28}H_{34}NO_{16}S_2$	259.0124, 223.0604, 205.0501, 190.0262, 96.9584, 79.9557, 74.9894	3,4,5-trimethoxybenzyl# or isomer	c
192	34.56	504.0835	-2.12	$C_{16}H_{26}NO_{13}S_2$	274.9903, 240.9991, 96.9583, 79.9558, 74.9892	6-hydroxyhexyl or isomer	b
193	34.66	451.0161	2.25	$C_{11}H_{19}N_2O_{11}S_3$	274.9902, 259.0127, 241.0017, 96.9585, 79.9558, 74.9893	(Cystein-S-yl)methyl or isomer	a
194	35.64	639.1157	-1.35	$C_{23}H_{31}N_2O_{15}S_2$	274.9901, 259.0124, 241.0019, 96.9584, 79.9555, 74.9892	(1-methoxy-1H-indolyl)methyl or isomer	d
195	35.97	598.1277	2.1	$C_{22}H_{32}NO_{14}S_2$	259.0121, 241.0017, 195.0325, 96.9585, 74.9892	3-phenylpropyl or isomer	d

# : Potential new GLSs，* : Confirmed by GSL standards,a: Conventional core GSLs, b: Malonyled GSLs, c: Sinapoyled GSLs, d: Glucosylated GSLs.

Supplementary Materials S2

Supplementary Materials S2.1 ***Source code for the Python "Deep Learning-Assisted MDF".***

```
import pandas as pd
import numpy as np
from sklearn.model_selection import train_test_split
from sklearn.preprocessing import MinMaxScaler
from sklearn.metrics import classification_report, confusion_matrix
from imblearn.combine import SMOTETomek
import torch
import torch.nn as nn
import torch.optim as optim
from torch.utils.data import DataLoader, TensorDataset
import matplotlib.pyplot as plt
import seaborn as sns


def load_and_preprocess_data(file_path, label_column, feature_columns):
    df = pd.read_csv(file_path)
    df['label_code'] = df[label_column].astype('category').cat.codes

    for col in feature_columns:
        df[col] = df[col].astype(float)

    df = df.dropna(subset=feature_columns)

    for col in feature_columns:
        df[f'{col}_integer_part'] = df[col].apply(lambda x: int(x))
```

```
        df[f'{col}_fractional_part'] = df[col].apply(
            lambda x: min(x - int(x), 1 - (x - int(x))) * 1000
        )

    X = df[
        [f'{col}_integer_part' for col in feature_columns] +
        [f'{col}_fractional_part' for col in feature_columns]
    ].values
    y = df['label_code'].values

    scaler = MinMaxScaler()
    X = scaler.fit_transform(X)

    return train_test_split(X, y, test_size=0.3, random_state=42), scaler, df


def resample_data(X_train, y_train):
    smote_tomek = SMOTETomek(random_state=42)
    X_resampled, y_resampled = smote_tomek.fit_resample(X_train, y_train)
    return X_resampled, y_resampled


class FocalLoss(nn.Module):
    def __init__(self, alpha=1, gamma=2, logits=True, reduce=True):
        super(FocalLoss, self).__init__()
        self.alpha = alpha
        self.gamma = gamma
        self.logits = logits
        self.reduce = reduce

    def forward(self, inputs, targets):
        if self.logits:
            BCE_loss = nn.functional.cross_entropy(inputs, targets, reduction='none')
        else:
            BCE_loss = nn.functional.binary_cross_entropy_with_logits(
                inputs, targets, reduction='none'
            )
        pt = torch.exp(-BCE_loss)
        F_loss = self.alpha * (1 - pt) ** self.gamma * BCE_loss

        return torch.mean(F_loss) if self.reduce else F_loss


class Net(nn.Module):
    def __init__(self, input_dim, output_dim):
        super(Net, self).__init__()
        self.fc1 = nn.Linear(input_dim, 512)
        self.bn1 = nn.BatchNorm1d(512)
        self.fc2 = nn.Linear(512, 256)
        self.bn2 = nn.BatchNorm1d(256)
        self.fc3 = nn.Linear(256, 128)
```

```
        self.bn3 = nn.BatchNorm1d(128)
        self.fc4 = nn.Linear(128, 64)
        self.bn4 = nn.BatchNorm1d(64)
        self.fc5 = nn.Linear(64, output_dim)
        self.relu = nn.ReLU()
        self.dropout = nn.Dropout(0.01)

    def forward(self, x):
        x = self.relu(self.bn1(self.fc1(x)))
        x = self.dropout(x)
        x = self.relu(self.bn2(self.fc2(x)))
        x = self.dropout(x)
        x = self.relu(self.bn3(self.fc3(x)))
        x = self.dropout(x)
        x = self.relu(self.bn4(self.fc4(x)))
        x = self.fc5(x)
        return x


def train_model(model, train_loader, criterion, optimizer, num_epochs=100):
    train_losses, test_losses = [], []
    train_accuracies, test_accuracies = [], []

    for epoch in range(num_epochs):
        model.train()
        epoch_train_loss = 0
        correct_train = 0
        total_train = 0

        for inputs, labels in train_loader:
            optimizer.zero_grad()
            outputs = model(inputs)
            loss = criterion(outputs, labels)
            loss.backward()
            optimizer.step()

            epoch_train_loss += loss.item()
            _, predicted = torch.max(outputs.data, 1)
            total_train += labels.size(0)
            correct_train += (predicted == labels).sum().item()

        train_loss = epoch_train_loss / len(train_loader)
        train_accuracy = correct_train / total_train
        train_losses.append(train_loss)
        train_accuracies.append(train_accuracy)

        model.eval()
        epoch_test_loss = 0
        correct_test = 0
        total_test = 0
```

```
        with torch.no_grad():
            for inputs, labels in test_loader:
                outputs = model(inputs)
                loss = criterion(outputs, labels)
                epoch_test_loss += loss.item()
                _, predicted = torch.max(outputs.data, 1)
                total_test += labels.size(0)
                correct_test += (predicted == labels).sum().item()

        test_loss = epoch_test_loss / len(test_loader)
        test_accuracy = correct_test / total_test
        test_losses.append(test_loss)
        test_accuracies.append(test_accuracy)

        print(
            f'Epoch [{epoch + 1}/{num_epochs}], '
            f'Train Loss: {train_loss:.4f}, Test Loss: {test_loss:.4f}, '
            f'Train Accuracy: {train_accuracy:.4f}, Test Accuracy: {test_accuracy:.4f}'
        )

    return train_losses, test_losses, train_accuracies, test_accuracies


def evaluate_model(model, test_loader, criterion):
    model.eval()
    epoch_test_loss = 0
    correct_test = 0
    total_test = 0
    all_labels, all_predictions = [], []

    with torch.no_grad():
        for inputs, labels in test_loader:
            outputs = model(inputs)
            loss = criterion(outputs, labels)
            epoch_test_loss += loss.item()
            _, predicted = torch.max(outputs.data, 1)
            total_test += labels.size(0)
            correct_test += (predicted == labels).sum().item()
            all_labels.extend(labels.numpy())
            all_predictions.extend(predicted.numpy())

    test_loss = epoch_test_loss / len(test_loader)
    test_accuracy = correct_test / total_test
    report = classification_report(
        all_labels, all_predictions, zero_division=0, output_dict=True
    )
    conf_matrix = confusion_matrix(all_labels, all_predictions)

    print(f'Test Loss: {test_loss:.4f}, Test Accuracy: {test_accuracy:.4f}')
    return report, conf_matrix, test_loss, test_accuracy
```

```
def plot_results(train_losses, test_losses, train_accuracies, test_accuracies,
                 report, conf_matrix, label_names):
    plt.figure(figsize=(12, 6))
    plt.subplot(1, 2, 1)
    plt.plot(train_losses, label='Train Loss')
    plt.plot(test_losses, label='Test Loss')
    plt.title('Loss over epochs')
    plt.xlabel('Epoch')
    plt.ylabel('Loss')
    plt.legend()

    plt.subplot(1, 2, 2)
    plt.plot(train_accuracies, label='Train Accuracy')
    plt.plot(test_accuracies, label='Test Accuracy')
    plt.title('Accuracy over epochs')
    plt.xlabel('Epoch')
    plt.ylabel('Accuracy')
    plt.legend()
    plt.show()

    plt.figure(figsize=(10, 6))
    sns.heatmap(pd.DataFrame(report).iloc[:-1, :].T,
                annot=True, cmap="YlGnBu")
    plt.title('Classification Report Heatmap')
    plt.show()

    plt.figure(figsize=(10, 6))
    sns.heatmap(conf_matrix, annot=True, fmt='d', cmap='Blues',
                xticklabels=label_names, yticklabels=label_names)
    plt.xlabel('Predicted Label')
    plt.ylabel('True Label')
    plt.title('Confusion Matrix')
    plt.show()


if __name__ == '__main__':
    file_path = 's111jj.csv'
    label_column = 'label'
    feature_columns = ['M-H', 'M-mh']

    (X_train, X_test, y_train, y_test), scaler, df = \
        load_and_preprocess_data(file_path, label_column, feature_columns)

    X_train_resampled, y_train_resampled = resample_data(X_train, y_train)

    X_train = torch.tensor(X_train_resampled, dtype=torch.float32)
    X_test = torch.tensor(X_test, dtype=torch.float32)
    y_train = torch.tensor(y_train_resampled, dtype=torch.long)
    y_test = torch.tensor(y_test, dtype=torch.long)
```

```
train_dataset = TensorDataset(X_train, y_train)
test_dataset = TensorDataset(X_test, y_test)

train_loader = DataLoader(train_dataset, batch_size=64, shuffle=True)
test_loader = DataLoader(test_dataset, batch_size=64, shuffle=False)

model = Net(input_dim=4, output_dim=2)

criterion = FocalLoss()
optimizer = optim.Adam(model.parameters(), lr=0.0001, weight_decay=0.01)

train_losses, test_losses, train_accuracies, test_accuracies = \
  train_model(model, train_loader, criterion, optimizer, num_epochs=100)

report, conf_matrix, test_loss, test_accuracy = \
  evaluate_model(model, test_loader, criterion)

label_names = df[label_column].astype('category').cat.categories
plot_results(train_losses, test_losses, train_accuracies,
        test_accuracies, report, conf_matrix, label_names)
```

Supplementary Materials S2.*2 **Source code for the Python "Automated Diagnostic Ion Screening System".***

```
import csv
import os
import pymzml
import pandas as pd
from tqdm import tqdm


def load_spectra(mzml_file):
  run = pymzml.run.Reader(mzml_file)
  spectra = {}
  for spectrum in run:
    spectra[spectrum.ID] = spectrum
  return spectra


def check_diagnostic_ions(diagnostic_ions, fragments,
max_intensity):
  diagnostic_ions_found = {}
  for diagnostic_ion in diagnostic_ions:
    mz = diagnostic_ion['mz']
    min_rel_abundance, max_rel_abundance =
diagnostic_ion['relative_abundance_range']
    max_peak_intensity = 0
    selected_peak_mz = None

    for peak in fragments:
```

```
            if abs(peak[0] - mz) <= 0.01:
                if peak[1] > max_peak_intensity:
                    max_peak_intensity = peak[1]
                    selected_peak_mz = peak[0]

        if selected_peak_mz is not None:
            relative_abundance = max_peak_intensity / max_intensity
if max_intensity > 0 else 0.0
            if min_rel_abundance <= relative_abundance <=
max_rel_abundance:
                diagnostic_ions_found[mz] = {
                    'intensity': max_peak_intensity,
                    'relative': relative_abundance
                }
                print(f"Found diagnostic ion {mz} with relative
abundance {relative_abundance}")
            else:
                diagnostic_ions_found[mz] = {
                    'intensity': 0,
                    'relative': 0
                }
                print(f"Diagnostic ion {mz} found but relative abundance
{relative_abundance} out of range")
        else:
            diagnostic_ions_found[mz] = {
                'intensity': 0,
                'relative': 0
            }
            print(f"Diagnostic ion {mz} not found")
    return diagnostic_ions_found


def find_diagnostic_ions_ms2(spectra, and_diagnostic_ions,
or_diagnostic_ions):
    results = []

    for spectrum in tqdm(spectra.values(), desc="Processing
spectra"):
        if spectrum.ms_level == 2:
            precursor = spectrum.selected_precursors[0]
            precursor_mz = precursor["mz"]
            precursor_charge = precursor.get("charge", 'N/A')

            retention_time = spectrum.scan_time_in_minutes() if 'scan
time' in spectrum else 'N/A'
            fragments = [(peak[0], peak[1]) for peak in
spectrum.peaks("centroided")]

            diagnostic_ions_result = {}
            max_intensity = max(peak[1] for peak in fragments) if
fragments else 1
```

```
            and_results = check_diagnostic_ions(and_diagnostic_ions,
fragments, max_intensity)
            or_results = check_diagnostic_ions(or_diagnostic_ions,
fragments, max_intensity)

            all_and_ions_valid = all(
              mz in and_results and and_results[mz]['relative'] >=
diagnostic_ion['relative_abundance_range'][0]
              for diagnostic_ion in and_diagnostic_ions for mz in
[diagnostic_ion['mz']]
            )

            if all_and_ions_valid:
              diagnostic_ions_result.update(and_results)
              diagnostic_ions_result.update(or_results)

              result = {
                'spectrum_id': spectrum.ID,
                'precursor_mz': precursor_mz,
                'precursor_charge': precursor_charge,
                'retention_time': retention_time,
                'fragments': fragments,
                'diagnostic_ions': diagnostic_ions_result
              }
              results.append(result)
              if len(results) % 100 == 0:
                print(f"Processed {len(results)} spectra")

        return results


    def save_results_to_excel(results, output_path,
and_diagnostic_ions, or_diagnostic_ions):
        dds_data = []
        dbs_data = []
        for result in results:
          fragment_ions = ", ".join([f"{fragment[0]:.4f}" for fragment in
result['fragments']])
          row = [
            result['spectrum_id'],
            result['precursor_mz'],
            result['precursor_charge'],
            result['retention_time'],
            fragment_ions,
          ]
          contains_precursor = any(
            abs(peak[0] - result['precursor_mz']) <= 0.01 for peak in
result['fragments']
          )
          for and_ion in and_diagnostic_ions:
```

```
            mz = and_ion['mz']
            if mz in result['diagnostic_ions']:
                relative_abundance =
result['diagnostic_ions'][mz]['relative']
                row.extend([mz, relative_abundance])
            else:
                row.extend([mz, 0])
        for or_ion in or_diagnostic_ions:
            mz = or_ion['mz']
            if mz in result['diagnostic_ions']:
                relative_abundance =
result['diagnostic_ions'][mz]['relative']
                row.extend([mz, relative_abundance])
            else:
                row.extend([mz, 0])
        if contains_precursor:
            dds_data.append(row)
        else:
            dbs_data.append(row)

    dds_df = pd.DataFrame(dds_data, columns=['Spectrum ID',
'Precursor m/z', 'Precursor Charge', 'Retention Time', 'Fragment Ions
m/z'] +
                          [f'Diagnostic Ion(AND) {and_ion["mz"]}'
for and_ion in and_diagnostic_ions] +
                          [f'Relative Intensity' for _ in
and_diagnostic_ions] +
                          [f'Diagnostic Ion(OR) {or_ion["mz"]}' for
or_ion in or_diagnostic_ions] +
                          [f'Relative Intensity' for _ in
or_diagnostic_ions])
    dbs_df = pd.DataFrame(dbs_data, columns=['Spectrum ID',
'Precursor m/z', 'Precursor Charge', 'Retention Time', 'Fragment Ions
m/z'] +
                          [f'Diagnostic Ion(AND) {and_ion["mz"]}'
for and_ion in and_diagnostic_ions] +
                          [f'Relative Intensity' for _ in
and_diagnostic_ions] +
                          [f'Diagnostic Ion(OR) {or_ion["mz"]}' for
or_ion in or_diagnostic_ions] +
                          [f'Relative Intensity' for _ in
or_diagnostic_ions])

    with pd.ExcelWriter(output_path) as writer:
        dds_df.to_excel(writer, sheet_name='dds', index=False)
        dbs_df.to_excel(writer, sheet_name='dbs', index=False)


def merge_precursors(df, ppm_tolerance):
    df['Precursor m/z'] = df['Precursor m/z'].astype(float)
    df = df.sort_values(by='Precursor m/z')
```

```
    merged_rows = []
    current_group = []

    for index, row in df.iterrows():
        if not current_group:
            current_group.append(row)
        else:
            mz_tolerance = row['Precursor m/z'] * ppm_tolerance / 1e6
            if abs(current_group[-1]['Precursor m/z'] - row['Precursor
m/z']) <= mz_tolerance:
                current_group.append(row)
            else:
                representative_row = merge_group(current_group)
                merged_rows.append(representative_row)
                current_group = [row]

    if current_group:
        representative_row = merge_group(current_group)
        merged_rows.append(representative_row)

    merged_df = pd.DataFrame(merged_rows)
    return merged_df


def merge_group(group):
    if len(group) == 1:
        return group[0].to_dict()

    representative_row = group[0].copy()
    for key in group[0].keys():
        if 'Diagnostic Ion' in key or 'Relative Intensity' in key:
            values = [pd.to_numeric(row[key], errors='coerce') for row
in group]
            values = [v for v in values if not pd.isnull(v) and v > 0]
            if values:
                representative_row[key] = max(values)
            else:
                representative_row[key] = 0
    return representative_row.to_dict()


def process_and_merge(input_excel, output_excel,
ppm_tolerance=5):
    df = pd.read_excel(input_excel, sheet_name=None)
    dds_df = df['dds']
    dbs_df = df['dbs']

    hbdds_df = merge_precursors(dds_df, ppm_tolerance)
    hbdbs_df = merge_precursors(dbs_df, ppm_tolerance)

    with pd.ExcelWriter(output_excel) as writer:
```

```
hbdds_df.to_excel(writer, sheet_name='hbdds', index=False)
hbdbs_df.to_excel(writer, sheet_name='hbdbs', index=False)


if __name__ == "__main__":
    mzml_file = ''
    and_diagnostic_ions = [
        {'mz': , 'relative_abundance_range': ()},
    ]
    or_diagnostic_ions = [
        {'mz':, 'relative_abundance_range': ()},
        {'mz':, 'relative_abundance_range': ()}
    ]
    output_excel = '

    spectra = load_spectra(mzml_file)
    results = find_diagnostic_ions_ms2(spectra,
and_diagnostic_ions, or_diagnostic_ions)
    save_results_to_excel(results, output_excel,
and_diagnostic_ions, or_diagnostic_ions)

    merged_output_excel = ''
process_and_merge(output_excel, merged_output_excel, ppm_tolerance=5)
```

Supplementary Materials S2.*3 **Source code for the Python "Tracer Molecular Networking".***

```
import os
import pandas as pd

class MGFSpectrum:
    def __init__(self):
        self.title = None
        self.pepmass = None
        self.charge = None
        self.mslevel = None
        self.feature_id = None
        self.scans = None
        self.rt_in_seconds = None
        self.merged_stats = None
        self.mz_values = []
        self.intensity_values = []

    def __str__(self):
        return f"Title: {self.title}\nPepMass: {self.pepmass}\nCharge:
{self.charge}\nMSLevel: {self.mslevel}\nPeaks: {len(self.mz_values)}"

def parse_mgf(file_path):
    spectra = []
    current_spectrum = None
```

```
    with open(file_path, 'r') as file:
        for line in file:
            line = line.strip()

            if line.startswith("BEGIN IONS"):
                current_spectrum = MGFSpectrum()
            elif line.startswith("END IONS"):
                spectra.append(current_spectrum)
                current_spectrum = None
            elif line.startswith("TITLE="):
                current_spectrum.title = line.split("=", 1)[1]
            elif line.startswith("PEPMASS="):
                pepmass_values = line.split("=", 1)[1].split()
                current_spectrum.pepmass = tuple(map(float,
pepmass_values))
            elif line.startswith("CHARGE="):
                current_spectrum.charge = line.split("=", 1)[1]
            elif line.startswith("MSLEVEL="):
                current_spectrum.mslevel = int(line.split("=", 1)[1])
            elif line.startswith("FEATURE_ID="):
                current_spectrum.feature_id = line.split("=", 1)[1]
            elif line.startswith("SCANS="):
                current_spectrum.scans = line.split("=", 1)[1]
            elif line.startswith("RTINSECONDS="):
                current_spectrum.rt_in_seconds = line.split("=", 1)[1]
            elif line.startswith("MERGED_STATS="):
                current_spectrum.merged_stats = line.split("=", 1)[1]
            else:
                try:
                    mz, intensity = map(float, line.split())
                    current_spectrum.mz_values.append(mz)
                    current_spectrum.intensity_values.append(intensity)
                except ValueError:
                    pass

    return spectra


def filter_spectra(spectra, target_mz, neutral_losses, tolerance_ppm,
min_pepmass):
    tolerance = tolerance_ppm * 1e-6
    filtered_spectra = []

    def check_matches(spectrum, targets):
        max_intensity = max(spectrum.intensity_values, default=1)
        return all(
            any(
                abs(mz - tmz[0]) / tmz[0] <= tolerance and (intensity /
max_intensity) >= tmz[1][0] and (intensity / max_intensity) <= tmz[1][1]
                for mz, intensity in zip(spectrum.mz_values,
spectrum.intensity_values)
```

```
                )
                for tmz in targets["and"]
            ) and any(
                any(
                    abs(mz - tmz[0]) / tmz[0] <= tolerance and (intensity / max_intensity) >= tmz[1][0] and (intensity / max_intensity) <= tmz[1][1]
                    for mz, intensity in zip(spectrum.mz_values, spectrum.intensity_values)
                )
                for tmz in targets["or"]
            )

        for spectrum in spectra:
            if spectrum.pepmass and spectrum.mslevel == 2 and spectrum.pepmass[0] >= min_pepmass:
                if check_matches(spectrum, target_mz) or check_matches(spectrum, neutral_losses):
                    filtered_spectra.append(spectrum)

        return filtered_spectra

    def merge_peaks(mz_values, intensity_values, tolerance_ppm):
        tolerance = tolerance_ppm * 1e-6
        merged_mz = []
        merged_intensity = []

        while mz_values:
            ref_mz = mz_values[0]
            ref_intensity = intensity_values[0]
            to_merge_indices = [i for i, mz in enumerate(mz_values) if abs(mz - ref_mz) / ref_mz <= tolerance]

            merged_mz.append(sum(mz_values[i] * intensity_values[i] for i in to_merge_indices) /
                             sum(intensity_values[i] for i in to_merge_indices))
            merged_intensity.append(sum(intensity_values[i] for i in to_merge_indices))

            for i in sorted(to_merge_indices, reverse=True):
                del mz_values[i]
                del intensity_values[i]

        return merged_mz, merged_intensity

    def merge_pepmass(spectra, tolerance_ppm):
        tolerance = tolerance_ppm * 1e-6
        grouped_spectra = []

        while spectra:
            ref_spectrum = spectra.pop(0)
            close_spectra = [ref_spectrum]
```

```
            for spectrum in spectra[:]:
                if abs(spectrum.pepmass[0] - ref_spectrum.pepmass[0]) /
ref_spectrum.pepmass[0] <= tolerance:
                    close_spectra.append(spectrum)
                    spectra.remove(spectrum)

            if len(close_spectra) > 1:
                merged_spectrum = MGFSpectrum()
                merged_spectrum.pepmass = (sum(s.pepmass[0] for s in
close_spectra) / len(close_spectra),
                                           sum(s.pepmass[1] for s in close_spectra if
len(s.pepmass) > 1) / len(close_spectra))
                merged_spectrum.charge = ref_spectrum.charge
                merged_spectrum.mslevel = ref_spectrum.mslevel
                merged_spectrum.feature_id = ref_spectrum.feature_id
                merged_spectrum.scans = ref_spectrum.scans
                merged_spectrum.rt_in_seconds = ref_spectrum.rt_in_seconds
                merged_spectrum.merged_stats = ref_spectrum.merged_stats
                for spectrum in close_spectra:
                    merged_spectrum.mz_values.extend(spectrum.mz_values)

merged_spectrum.intensity_values.extend(spectrum.intensity_values)

                merged_spectrum.mz_values,
merged_spectrum.intensity_values = merge_peaks(
                    merged_spectrum.mz_values,
merged_spectrum.intensity_values, tolerance_ppm)
                grouped_spectra.append(merged_spectrum)
            else:
                grouped_spectra.append(ref_spectrum)

        return grouped_spectra

    def export_filtered_mgf(filtered_spectra, output_file_path):
        with open(output_file_path, 'w') as file:
            for spectrum in filtered_spectra:
                file.write("BEGIN IONS\n")
                if spectrum.feature_id:
                    file.write(f"FEATURE_ID={spectrum.feature_id}\n")
                if spectrum.title:
                    file.write(f"TITLE={spectrum.title}\n")
                if spectrum.pepmass:
                    pepmass_str = f"{spectrum.pepmass[0]:.6f}"
                    if len(spectrum.pepmass) == 2 and spectrum.pepmass[1] != 0:
                        pepmass_str += f" {spectrum.pepmass[1]:.6f}"
                    file.write(f"PEPMASS={pepmass_str}\n")
                if spectrum.scans:
                    file.write(f"SCANS={spectrum.scans}\n")
                if spectrum.rt_in_seconds:
                    file.write(f"RTINSECONDS={spectrum.rt_in_seconds}\n")
```

```
            if spectrum.charge:
                file.write(f"CHARGE={spectrum.charge}\n")
            if spectrum.mslevel:
                file.write(f"MSLEVEL={spectrum.mslevel}\n")
            if spectrum.merged_stats:
                file.write(f"MERGED_STATS={spectrum.merged_stats}\n")

            for mz, intensity in zip(spectrum.mz_values, spectrum.intensity_values):
                file.write(f"{mz:.4f} {intensity:.1E}\n".replace('+', ''))
            file.write("END IONS\n")

mgf_file_path =
csv_file_path =
filtered_mgf_file_path =
filtered_csv_file_path =

mgf_spectra = parse_mgf(mgf_file_path)

target_mz = {
    "and": [

    ],
    "or": [

    ]
}

neutral_losses = {
    "and": [

    ],
    "or": [


    ]
}

tolerance_ppm = 10
min_pepmass =

filtered_spectra = filter_spectra(mgf_spectra, target_mz, neutral_losses, tolerance_ppm, min_pepmass)

merged_spectra = merge_pepmass(filtered_spectra, tolerance_ppm)

export_filtered_mgf(merged_spectra, filtered_mgf_file_path)

filtered_feature_ids = [spectrum.feature_id for spectrum in merged_spectra if spectrum.feature_id]
```

```
quant_data = pd.read_csv(csv_file_path)

filtered_quant_data = quant_data[quant_data['row ID'].astype(str).isin(filtered_feature_ids)]

filtered_quant_data.to_csv(filtered_csv_file_path, index=False)

print("MGF and CSV files filtered and exported successfully!")
```

Supplementary Materials S2.*4* ***Source code for the Python "Substituent Combination Algorithm".***

```
import multiprocessing
from itertools import combinations_with_replacement
import re
from collections import defaultdict
import pandas as pd
import time

substituents = {
    "CH3": 15.02347509,
    "OCH3": 31.01838971,
    "CH2": 14.01565006,
    "CH": 13.00782503,
    "C2": 24.00000,
    "C2H": 25.00782503,
    "C2H2": 26.01565006,
    "C2H3": 27.02347509,
    "HSO3": 80.96463989,
    "SO2": 63.96190024,
    "SO": 47.96698562,
    "S": 31.97207100,
    "OH": 17.00273965,
    "O": 15.99491462,
    "CO": 27.99491462,
    "C6H5": 77.03912515,
    "C6H4": 76.03130012,
    "C6H3": 75.02347509,
    "C6H2": 74.01565006,
    "C8H6N": 116.05002418,
    "C8H5N": 115.04219915,
    "C8H4N": 114.03437412,
    "C8H3N": 113.02654909,
    "C8H2N": 112.01872406,
    "NH": 15.01089903,
}

base_molecule = {
    "formula": ,
    "molar_mass":
```

```
}

tolerance_ppm = 10

def parse_formula(formula):
    elements = re.findall(r'([A-Z][a-z]*)(\d*)', formula)
    parsed_formula = defaultdict(int)
    for element, count in elements:
        parsed_formula[element] += int(count) if count else 1
    return parsed_formula

def combine_formulas(base_formula, substituent_formulas):
    combined_formula = parse_formula(base_formula)
    for formula in substituent_formulas:
        for element, count in parse_formula(formula).items():
            combined_formula[element] += count
    return combined_formula

def formula_to_string(formula_dict):
    return ''.join(f"{element}{count if count > 1 else ''}" for element, count
in sorted(formula_dict.items()))

def is_valid_formula(formula_dict):
    return True

def find_substituent_combinations(base, substituents, target_mass,
tolerance_ppm):
        base_mass = base["molar_mass"]
        substituent_masses = list(substituents.values())
        substituent_names = list(substituents.keys())

        ending_groups = {"CH3", "OCH3", "HSO3", "OH", "C6H5",
"C8H6N", "C2H3"}
        missing_groups = {
            "C6H4": 1, "C6H3": 2, "C6H2": 3, "C8H5N": 1, "CH": 2,
"C2H2": 1,
            "C2H": 2, "C2": 3, "C8H4N": 2, "C8H3N": 3, "C8H2N": 4
        }

        for r in range(1, len(substituents) + 1):
            for combination in
combinations_with_replacement(substituent_masses, r):
                substituent_formulas =
[substituent_names[substituent_masses.index(mass)] for mass in
combination]
                total_mass = sum(combination) + base_mass
                error_ppm = abs((total_mass - target_mass) / target_mass) * 10
** 6
                if error_ppm <= tolerance_ppm:
                    combined_formula_dict = combine_formulas(base["formula"],
substituent_formulas)
```

```
                missing_count = sum(1 for group in substituent_formulas if group in missing_groups)
                ending_count = sum(1 for group in substituent_formulas if group in ending_groups)

                valid_combination = False
                if missing_count == 0 and ending_count == 1:
                    valid_combination = True
                elif missing_count > 0:
                    required_ending_count = sum(missing_groups[group] for group in substituent_formulas if group in missing_groups) + 1 - missing_count
                    if ending_count == required_ending_count:
                        valid_combination = True

                if valid_combination and is_valid_formula(combined_formula_dict):
                    yield substituent_formulas

def process_row(target_molar_mass, results_list, last_output_time, max_output_wait_time, output_csv_file_path):
    try:
        for combo in find_substituent_combinations(base_molecule, substituents, target_molar_mass, tolerance_ppm):
            substituent_formulas = combo
            combined_formula_dict = combine_formulas(base_molecule["formula"], substituent_formulas)
            final_formula = formula_to_string(combined_formula_dict)
            results_list.append({
                "target_molar_mass": target_molar_mass,
                "substituent_formulas": substituent_formulas,
                "final_formula": final_formula,
                "error": ""
            })
            last_output_time.value = time.time()
            save_results_to_csv(results_list, output_csv_file_path)
    except Exception as e:
        results_list.append({
            "target_molar_mass": target_molar_mass,
            "substituent_formulas": [],
            "final_formula": "",
            "error": str(e)
        })
        save_results_to_csv(results_list, output_csv_file_path)

def save_results_to_csv(results_list, output_csv_file_path):
    results = list(results_list)
    output_df = pd.DataFrame(results)
    output_df.to_csv(output_csv_file_path, index=False)
```

```
def main(max_output_wait_time):
    csv_file_path =
    df = pd.read_csv(csv_file_path)

    output_csv_file_path =

    manager = multiprocessing.Manager()
    results_list = manager.list()
    last_output_time = manager.Value('d', time.time())

    for index, row in df.iterrows():
        target_molar_mass = row["Precursor m/z"]
        print(f"Processing target molar mass: {target_molar_mass}")
        last_output_time.value = time.time()
        process = multiprocessing.Process(target=process_row,
args=(target_molar_mass, results_list, last_output_time,
max_output_wait_time, output_csv_file_path))
        process.start()

        process.join(max_output_wait_time)
        if time.time() - last_output_time.value > max_output_wait_time:
            print(f"Target molar mass: {target_molar_mass} did not produce
output within {max_output_wait_time} seconds, terminating process")
            process.terminate()
            process.join()
            results_list.append({
                "target_molar_mass": target_molar_mass,
                "substituent_formulas": [],
                "final_formula": "",
                "error": "Execution time exceeded limit"
            })
            save_results_to_csv(results_list, output_csv_file_path)

if __name__ == '__main__':
main(max_output_wait_time=100)
```

Supplementary Materials S2.5 ***Source code for the Python "PubChem Compound Search".***

```
import pandas as pd
import requests
import time
def get_compound_info_by_formula(formula):
    base_url =
"https://pubchem.ncbi.nlm.nih.gov/rest/pug/compound"
    url = f"{base_url}/formula/{formula}/JSON"
    results = []
    try:
        response = requests.get(url)
        response.raise_for_status()
```

```
            data = response.json()
            # Check if we need to wait for the result
            if 'Waiting' in data:
                list_key = data['Waiting']['ListKey']
                poll_url = f"{base_url}/listkey/{list_key}/JSON"
                print(f"Waiting for the result... ListKey: {list_key}")
                while True:
                    poll_response = requests.get(poll_url)
                    poll_response.raise_for_status()
                    poll_data = poll_response.json()
                    if 'PC_Compounds' in poll_data:
                        compounds = poll_data['PC_Compounds']
                        for compound in compounds[:5]:  # Limit to 5 results
                            results.append(extract_compound_info(compound))
                        return results
                    elif 'Waiting' in poll_data:
                        print("Still waiting...")
                        time.sleep(5)  # Poll every 5 seconds
                    else:
                        print("No compounds found.")
                        return results
            compounds = data.get('PC_Compounds', [])
            if not compounds:
                print("No compounds found.")
                return results
            for compound in compounds[:5]:  # Limit to 5 results
                results.append(extract_compound_info(compound))
        except requests.RequestException as e:
            print(f"Request failed: {e}")
        return results
    def extract_compound_info(compound):
        result = {}
        cid = compound.get('id', {}).get('id', {}).get('cid', 'N/A')
        result['Exact Mass'] = next(
            (prop.get('value',   {}).get('sval',   'N/A')   for   prop   in
compound.get('props', []) if
             prop.get('urn', {}).get('label') == 'Mass'), 'N/A')
        result['Charge'] = compound.get('charge', 'N/A')
        result['IUPAC Name'] = next(
            (prop.get('value',   {}).get('sval',   'N/A')   for   prop   in
compound.get('props', []) if
             prop.get('urn', {}).get('label') == 'IUPAC Name'), 'N/A')
        result['Names'] = get_all_names_from_pubchem(cid)
        return result

    def get_all_names_from_pubchem(cid):
        base_url                                                          =
f"https://pubchem.ncbi.nlm.nih.gov/rest/pug/compound/cid/{cid}/syn
onyms/JSON"
        try:
            response = requests.get(base_url)
```

```
        response.raise_for_status()
        data = response.json()
        # Extract and return all names
        synonyms = data.get('InformationList', {}).get('Information', [])
        if synonyms:
            names = synonyms[0].get('Synonym', [])
            return ', '.join(names)
        else:
            return None

    except requests.RequestException as e:
        print(f"Request failed for CID {cid}: {e}")
        return None
def process_csv(input_csv, output_csv):
    df = pd.read_csv(input_csv)
    result_rows = []
    for index, row in df.iterrows():
        formula = row['final_formula']
        m_h_formula = row['M+H']
        if pd.notna(formula):
            info_list = get_compound_info_by_formula(formula)
            for info in info_list:
                result_row = {
                    'Original Formula': formula,
                    'Exact Mass': info['Exact Mass'],
                    'Charge': info['Charge'],
                    'IUPAC Name': info['IUPAC Name'],
                    'Names': info['Names']
                }
                result_rows.append(result_row)
        if pd.notna(m_h_formula):
            info_list = get_compound_info_by_formula(m_h_formula)
            for info in info_list:
                result_row = {
                    'Original Formula': m_h_formula,
                    'Exact Mass': info['Exact Mass'],
                    'Charge': info['Charge'],
                    'IUPAC Name': info['IUPAC Name'],
                    'Names': info['Names']
                }
                result_rows.append(result_row)
    result_df = pd.DataFrame(result_rows)
    result_df.to_csv(output_csv, index=False)
# Replace 'input.csv' with your actual CSV file name
input_csv =.
output_csv =
process_csv(input_csv, output_csv)
```
